# Phenotype Distinctions in Mice Deficient in the Neuron-Specific α3 Subunit of Na,K-ATPase: *Atp1a*3^tm1Ling/+^ and *Atp1a3*^+/D801Y^

**DOI:** 10.1523/ENEURO.0101-24.2024

**Published:** 2024-08-20

**Authors:** Yi Bessie Liu, Elena Arystarkhova, Amanda N. Sacino, Margit V. Szabari, Cathleen M. Lutz, Markus Terrey, Natalia S. Morsci, Tatjana C. Jakobs, Karin Lykke-Hartmann, Allison Brashear, Elenora Napoli, Kathleen J. Sweadner

**Affiliations:** ^1^Department of Neurosurgery, Massachusetts General Hospital, Boston, Massachusetts 02114; ^2^Harvard Medical School, Boston, Massachusetts 02115; ^3^Department Anesthesia, Massachusetts General Hospital, Boston, Massachusetts 02114; ^4^The Jackson Laboratory, Bar Harbor, Maine 04609; ^5^Department of Ophthalmology, Massachusetts Eye and Ear Infirmary/Schepens Eye Research Institute, Harvard Medical School, Boston, Massachusetts 02114; ^6^Department of Biomedicine, Aarhus University, Aarhus 8000, Denmark; ^7^Department of Neurology, Jacobs School of Medicine and Biomedical Sciences, University at Buffalo, Buffalo, New York 14203; ^8^Department of Neurology, University of California Davis School of Medicine, Sacramento, California 95817

**Keywords:** *ATP1A3*, disease mutation, motor testing, mouse model, Na,K-ATPase

## Abstract

*ATP1A3* is a Na,K-ATPase gene expressed specifically in neurons in the brain. Human mutations are dominant and produce an unusually wide spectrum of neurological phenotypes, most notably rapid-onset dystonia parkinsonism (RDP) and alternating hemiplegia of childhood (AHC). Here we compared heterozygotes of two mouse lines, a line with little or no expression (*Atp1a*3^tm1Ling/+^) and a knock-in expressing p.Asp801Tyr (D801Y, *Atp1a3*^+/D801Y^). Both mouse lines had normal lifespans, but *Atp1a3*^+/D801Y^ had mild perinatal mortality contrasting with D801N mice (*Atp1a3*^+/D801N^), which had high mortality. The phenotypes of *Atp1a*3^tm1Ling/+^ and *Atp1a3*^+/D801Y^ were different, and testing of each strain was tailored to its symptom range. *Atp1a*3^tm1Ling/+^ mice displayed little at baseline, but repeated ethanol intoxication produced hyperkinetic motor abnormalities not seen in littermate controls. *Atp1a3*^+/D801Y^ mice displayed robust phenotypes: hyperactivity, diminished posture consistent with hypotonia, and deficiencies in beam walk and wire hang tests. Symptoms also included qualitative motor abnormalities that are not well quantified by conventional tests. Paradoxically, *Atp1a3*^+/D801Y^ showed sustained better performance than wild type on the accelerating rotarod. *Atp1a3*^+/D801Y^ mice were overactive in forced swimming and afterward had intense shivering, transient dystonic postures, and delayed recovery. Remarkably, *Atp1a3*^+/D801Y^ mice were refractory to ketamine anesthesia, which elicited hyperactivity and dyskinesia even at higher dose. Neither mouse line exhibited fixed dystonia (typical of RDP patients), spontaneous paroxysmal weakness (typical of AHC patients), or seizures but had consistent, measurable neurological abnormalities. A gradient of variation supports the importance of studying multiple *Atp1a3* mutations in animal models to understand the roles of this gene in human disease.

## Graphical abstract

**Figure:**
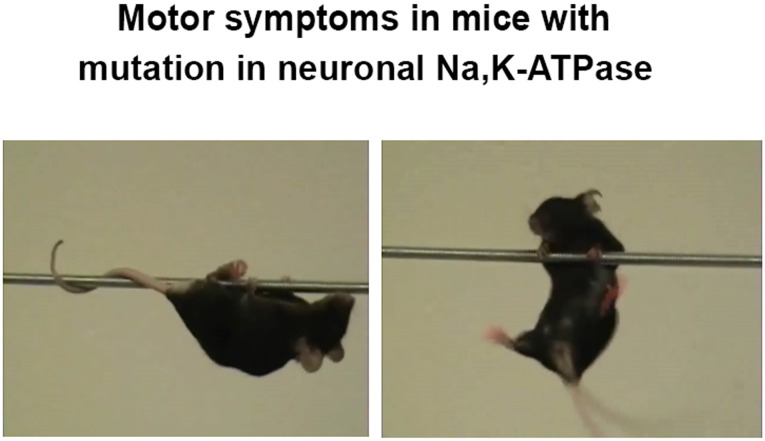
Visual Abstract

## Significance Statement

Different dominant mutations in the neuronal Na,K-ATPase cause an unusually wide range of symptoms in humans. *Atp1a3* mouse models also differ greatly in mortality and in visible impairments, but conventional motor tests do not capture their manifestations very well. Two models were compared here with conventional, modified, and novel methods with some surprising results.

## Introduction

The Na,K-ATPase catalyzes the active transport of Na^+^ and K^+^ ions across the plasma membrane, and because it is electrogenic, it directly affects the membrane potential. Its functional roles in the nervous system are more complex than those of many ion channels (Benarroch, 2011). The Na,K-ATPase is composed of three subunits, alpha (α), beta (β) and gamma (γ). The isoforms of its catalytic α subunit in the brain have different cell type and developmental expression patterns (McGrail et al., 1991; Watts et al., 1991; Herrera et al., 1994; Moseley et al., 2003): the α1 isoform is expressed in many but not all neurons and in some astrocytes; the α2 isoform is the predominant form in astrocytes but also in neurons in the perinatal period; and the α3 isoform is expressed in a majority of neurons, and for some types of neurons, such as in the basal ganglia and the inhibitory neurons of the cerebellum, it is the predominant form.

The α3 isoform is thought to have kinetic properties adapted for large shifts in intracellular Na^+^, the rate-limiting substrate for pump activity (Dobretsov and Stimers, 2005; Azarias et al., 2013). Inhibitor studies reveal isoform-specific effects on neuronal electrophysiology, and the Na,K-ATPases also have many cell biological interactions with important CNS proteins [reviewed in Dobretsov and Stimers (2005); Benarroch (2011)].

In humans, mutations in the *ATP1A3* gene, which encodes the α3 isoform, are associated with several rare neurological disorders; rapid-onset dystonia parkinsonism (RDP; de Carvalho Aguiar et al., 2004); alternating hemiplegia of childhood (AHC; Heinzen et al., 2012; Rosewich et al., 2012); cerebellar ataxia, areflexia, pes cavus, optic atrophy, and sensorineural hearing loss syndrome (CAPOS; Demos et al., 2014); and relapsing encephalopathy with cerebellar ataxia (RECA; Dard et al., 2015), also known as fever-induced paroxysmal weakness and encephalopathy (FIPWE; Yano et al., 2017). In addition, there are severe pre- and perinatal presentations, including brain malformations, intractable epilepsy, apnea, hypotonia, cerebellar hypoplasia with dystonia and encephalopathy, and developmental delay (Vezyroglou et al., 2022). All of the above can be caused by de novo missense mutations in *ATP1A3*. RDP carriers show few symptoms before a lifetime of dystonia is triggered, often by stressful events at any age (Brashear et al., 2007; Haq et al., 2019). AHC is characterized by transient hypotonia or dystonia with onset before 18 months of age. CAPOS and RECA/FIPWE are triggered by fevers but have different consequences. All these phenotypes have emerged since the initial report of *ATP1A3* mutations causing RDP in 2004 (de Carvalho Aguiar et al., 2004). We now know that a wide spectrum of overlapping symptoms can be traced to *ATP1A3* mutations in patients (Salles et al., 2021; Vezyroglou et al., 2022).

Stresses, whether mild in the case of AHC, severe in RDP, or associated with febrile infections in CAPOS or FIPWE, play a role in all postnatal-onset *ATP1A3* syndromes. It is enticing to hypothesize that stressful events make demands on pump activity that cannot be met by cells with impaired mutant enzyme, but the neuroendocrine consequences of stress may have deleterious effects in neurons with reduced α3 Na,K-ATPase (Dos-Santos et al., 2023), and temperature sensitivity of mutant proteins can also play a role (Arystarkhova et al., 2023).

Symptomatic animal models are needed. There are now several mouse models, and they have advanced our knowledge of the phenotypic presentation of mutations causing *ATP1A3*-related disorders [for review see Ng et al. (2021)]. Previous findings have shown that knock-out heterozygous mice (*Atp1a3^+/−^*) exhibit deficits in spatial learning, hyperactivity, and enhanced locomotor activity in response to amphetamine or intracranial kainate administration (Ikeda et al., 2013), but no lasting symptoms of dystonia (Moseley et al., 2007), and that a knock-in of one of the RDP/AHC mutations, D801Y (*Atp1a3*^+/D801Y^*; Atp1a3*^tm1.1Tmklh^; MGI 6163502), led to the development of features characteristic of both AHC and RDP (Holm et al., 2016; Isaksen et al., 2017). Similarly, knock-in of one of the AHC mutations, D801N (*Atp1a3*^+/D801N^; *Mashl*^+/−^; MGI 6162645), recapitulated many of the clinical features of AHC, including hemiplegia, dystonia, tremor, and seizures, as well as deficits in memory, gait, and motor coordination and control (Hunanyan et al., 2015).

In the present study, we focus on the phenotypic characteristics of two models: *Atp1a*3^tm1Ling/+^ as a model of a “loss-of-function allele” as defined in genetics (meaning loss of expression due to truncation or deletion) and *Atp1a3*^+/D801Y^ as a model of intermediate phenotype. Neither of these types of mutation are common in the clinical literature, possibly because they are underdiagnosed. Adult mice were examined for Atp1a3 isoform expression, and activity of Na,K-ATPase was tested under conditions that measure maximal enzyme function. Motor testing parameters were expanded to better define symptoms.

## Materials and Methods

### Mice

*Atp1a*3^tm1Ling/+^ mice were constructed as described previously [Moseley et al., 2007; designated *Atp1a3*^tm1Ling^ in the Mouse Genome Informatics (MGI) database; 3696954, and α3^+/KOI4^ in older sources] and were provided by Dr. A. Moseley and Dr. J.B. Lingrel, University of Cincinnati.

The Lingrel heterozygote mice had previously been tested on the 129/Black Swiss background (Moseley et al., 2007), on C57BL/6 (unspecified; DeAndrade et al., 2011), or on C57BL/6NCrl (Kirshenbaum et al., 2011b), but here were bred onto the C57BL/6NCrl background (Charles River Laboratories). *Atp1a3*^+/D801Y^ mice (*Atp1a3*^tm1.Tmklh^) were constructed as described previously (Holm et al., 2016). They were originally tested on the C57BL/6JRj (Janvier) background (Holm et al., 2016) or C57BL/6J (Isaksen et al., 2017), but here were bred onto C57BL/6NCrl. Breeding survival data were obtained from *Atp1a3*^+/D801N^ mice (C57BL/6J-*Atp1a3*^em3Lutzy^/*Mmjax*; MGI #6438218), generated at the Jackson Laboratory (MMRRC stock #66968). Both males and females were used for our experiments. Unless otherwise noted, data from the two sexes were pooled due to lack of a statistically significant effect of sex across outcomes. All procedures were approved by the institutional animal care and use committee of the Massachusetts General Hospital (*Atp1a*3^tm1Ling/+^ and *Atp1a3*^+/D801Y^) or the Jackson Laboratory (*Atp1a3*^+/D801N^).

### New insight into the Lingrel *Atp1a*3^tm1Ling/+^ mice

The mice were a by-product of an α3 knock-out attempt and contain a point mutation in *Atp1a3* intron 4, adjacent to the exon–intron splice site, which led to homozygous postnatal lethality. It resulted in aberrant splicing, adding 126 bp to the transcript. The aberrant transcript did not produce a protein product (Moseley et al., 2007), and notably, the mouse did not complement the I810N (*Myk*) line when hybridized (Clapcote et al., 2009).

A closer look at the sequence in light of later crystal structures of Na,K-ATPase sheds new light on the mutation's likely consequences. In the original report, an mRNA the same size as WT mRNA was seen in a −/− prenatal preparation (Moseley et al., 2007), which could lead to the conclusion that the alteration is not in fact a null allele. When the base adjacent to the exon (the G of GT, the splice donor) is mutated, another GT can sometimes be used as a splice donor. The one demonstrably used, 126 bp downstream, keeps the sequence in-frame; however the retained intron contains four in-frame stop codons, so a longer protein would not be possible. That longer mRNA was detected in −/− fetuses but not in heterozygotes (Moseley et al., 2007), suggesting that if the neurons expressing it are healthy enough, it is degraded by nonsense-mediated decay. Any other alternative GT splice donor has to be close enough for the gel mobility of the mRNA to be indistinguishable [Moseley et al. (2007), their Fig. 1*B*], and the closest one is an in-frame GT in the intron just two codons away. This would insert two amino acids, Val-Ser, at the junction of the first extracellular loop (L1–2) and the second membrane span (M2) in crystal structures. The insertion of two amino acids should either compromise the ion path entrance by distorting L1–2 and/or change the angle and rotation of M2, which should interfere with folding and activity. Consequently, this transcript is unlikely to produce active Na,K-ATPase, although that would have to be tested. The next closest potential splice donor GT would insert 10 amino acids, and upstream sites are much farther away.

### Genotyping

Mice were genotyped at weaning by PCR (REDExtract-N-Amp PCR kit, Sigma) using ear punches that also marked individuals for identification. The primers were as follows: *Atp1a3*^tm1.1Ling/^, (forward primer 5′TCCCTGCAGCCTCCTAAGTC; reverse primer 5′CTGCTATTTAGCCTAGGCTG); *Atp1a3*^+/D801Y^ (forward primer: 5′GCTTAAAGCACGGGACAAGA; reverse primer 5′GGAAGCCAGTGATTGGTTGT). *Atp1a3*^+/D801N^ mice were genotyped by real-time PCR (forward primer: 5′CTC TTG GCA CCA TCA CCA TC; reverse primer: 5′TTT AGT AGC AGC CAG GCT TAC C; WT probe: 5′CTG CAT TGA CCT GGG TAC C; mutant probe: 5′TCT GCA TTA ACC TAG GTA CCG AC). Male and female mice were included in all experiments in approximately equal numbers.

### Brain preparations

Brain samples were dissected as follows. Freshly removed brains were kept in ice-cold Dulbecco's PBS without Ca^2+^ or Mg^2+^. The brain was trisected immediately rostral to the hippocampus and again immediately rostral to the cerebellum. The cortex sample used was the portion lying over the hippocampus, and the hippocampus was dissected intact, taking care to remove any adherent choroid plexus (a rich source of α1). The cerebellum was lifted off the brainstem, and the fourth ventricle choroid plexus was removed before dissecting at the cerebellar peduncles. Together these regions are representative of mixed gray and white matter but relatively low in pure white matter tracts that contribute a disproportionate amount of lipid to the assays.

Crude homogenates of mouse brain samples were used to avoid the large losses that occur with conventional nuclear and mitochondrial centrifugation steps. Homogenates were in 315 mM sucrose, 20 mM Tris, 1 mM EDTA, pH 7.4, containing Roche complete mini protease inhibitor cocktail (1 tablet/25 ml buffer), and Sigma phosphatase inhibitor cocktail 1 (for serine/threonine phosphatases) at 1:100 dilution. Protein concentrations were determined with either the Lowry or the Pierce BCA protein assays. Homogenates were frozen in liquid nitrogen and stored at −70°C.

### Detection of Na,K-ATPase subunits

Gel electrophoresis and Western blots were performed using Invitrogen NuPAGE 4–12% MES gels and transferred onto nitrocellulose membranes. Antibodies for α3 were monoclonal antibody XVI-F9G10 (Affinity BioReagents; also known as MA3-915) or goat antiserum Na,K-ATPase α3 C16 (Santa Cruz Biotechnology). Loading controls were either anti-actin or anti-GAPDH or total proteins stained with Ponceau S. Signals were developed with West Dura luminol reagent (Thermo Fisher Scientific) and measured with a GE Healthcare LAS 4000 imaging system and ImageQuant software.

### Assay of Na,K-ATPase activity

The activity assay measured the hydrolysis of ATP in the test tube after pretreatment of the samples with SDS at a final detergent: protein ratio of 0.58. The Na,K-ATPase is resistant to denaturing effects of SDS at this ratio, although activity is lost at ratios above 0.8. We used a Na,K-ATPase assay in which BSA was used to buffer the SDS in order to control the exposure to detergent (Sweadner, 2016). In brief, brain sample homogenates were preincubated with SDS for 10 min at room temperature in a solution containing 2.0 mg/ml BSA, then diluted with three volumes of a solution containing 0.3 mg/ml BSA and no additional detergent, and kept on ice until assay. The ATPase reaction mixture contained (in mM) 140 NaCl, 20 KCl, 4 MgCl_2_, 3 disodium ATP (Sigma, vanadate-free), 30 histidine, pH 7.2, and it was prewarmed at 37°C. Samples containing 13.5 μg protein were added to 400 μl of aliquots of reaction mixture and incubated for 15 min, followed by quenching with acid molybdate and color development with Fiske–Subbarow reducing solution. Developed color was read at 700 nm with a spectrophotometer. Na,K-ATPase activity was defined as activity inhibited by ouabain. With this SDS treatment, almost all of the ouabain-insensitive background of ATP hydrolysis was inhibited. Because mouse α1 Na,K-ATPase has a low affinity for ouabain, we resolved the activity due to α3 plus α2 as that inhibited by 10 μM ouabain and activity due to α1 as that active in 10 μM ouabain but inhibited by 3 mM ouabain. The cutoff was determined empirically: ATPase activity in mouse kidney (α1) and mouse axolemma preps (α3, very depleted of α1 and α2) was measured with different concentrations of ouabain; there was still 15–20% activity left in the axolemma prep with 3 μM ouabain, while 10 μM showed complete inhibition. No difference for α1-dependent activity was observed at those concentrations. Total ouabain-sensitive specific activities were typically 60 μmol/h/mg protein, higher than obtained in similar protocols using milder detergents.

Retinal ganglion cell quantification was performed as reported previously (R. Wang et al., 2017). 

### Behavioral testing

Behavioral testing was done on the two strains independently except where noted. Testing was done during daylight hours.

#### *Atp1a*3^tm1Ling/+^ mice only

##### Motor tests

Grip strength was tested by the time to fall from an inverted cage top. The rack was shaken laterally three times to make them grip it well and inverted 30 cm above a padded surface. The time spent clinging to (or climbing around on) the rack was recorded, and the test was terminated after 60 s. Voluntary running was assessed using Bio-Serv plastic wheels on Teflon axles, equipped with magnets and meters that recorded both the distance run and the number of minutes spent running per 24 h period (De Bono et al., 2006). A minimum of six age- and sex-matched mice were tested for each group.

##### Ethanol vapor chamber

Attempts to administer ethanol in drinking water produced only increased ambulatory activity. An ethanol vapor protocol was followed because the objective was to produce deep intoxication culminating in loss of consciousness in an effort to mimic human binge drinking (Barbano et al., 2012). A vapor chamber was constructed from a rat cage (Rogers et al., 1979). An aquarium pump was measured to deliver 7.7 L of air per minute. Ethanol delivered by a calibrated peristaltic pump was introduced to the air stream in an evaporation flask heated by a glass bead bath. The vaporized ethanol then passed through the air intake port of the cage and escaped through the filter-topped lid. The lid was 90% covered by aluminum foil to direct the flow of air to the opposite end of the cage. A mouse's sensitivity to ethanol vapor is affected by weight, and so larger mice were tested separately from smaller, and ethanol flow rates were adjusted to produce strong intoxication within 3–5 h. The concentration of ethanol required was 10–25 mg/L of air. Mice in groups of eight were treated for 6 h a day 5 d a week for 2 or more weeks, spending the rest of the time in their home cages. The mice were not left unattended in ethanol vapor.

#### *Atp1a*3^tm1Ling/+^ and *Atp1a3*^+/D801Y^ mice

##### Activity chamber

For activity analysis, we used an OPTO-Varimex Minor activity meter (Columbus Instruments) to monitor individual activity by optical beam breaks, using an empty rat cage (37 × 25 × 19 cm) as the chamber. Mice were introduced to the activity chamber as a novel environment, and beam breaks were recorded for the time lengths noted.

##### Elevated beam

Mice were trained on 1 day and tested on the next. The beam used was a 150-cm-long, 9 mm cylindrical wood rod 24″ above a padded surface, and the goal was either the home cage or a dark enclosure. The tests were filmed. Mouse crossing time was recorded, and foot slips were counted by video review.

##### Rotarod

The accelerating rotarod test (Rotamex, Columbus Instruments) entailed accelerating the rod from 4 to 40 rpm over 180 s. Mice were trained for 2 d, two trials per day, with a 5 min break between trials. The results of two trials were recorded on the third day.

##### Forced swimming

Forced swimming was used as a stressor by adaptation of Porsolt test conditions (Porsolt et al., 1977; David et al., 2003), a widely used procedure for evaluating the behavioral effects of drugs used to treat depression. The water was 10–15 cm deep and 25°C except as noted, and the time was 15 min. Periods of swimming were terminated early if any mouse's nose was in the meniscus; this occurred occasionally with *Atp1a3*^+/D801Y^ mice apparently because of fatigue due to continuous swimming. Swimming was filmed and evaluated for time spent actively swimming. After swim, excess water in the fur was reduced by a brief squeeze in an absorbent paper towel, and mouse recovery was monitored in a tray until moving normally.

##### Ketamine treatment

For ketamine experiments, all mice had open-field activity testing to assess their baseline behavior, and so the activity chamber was technically no longer novel when on the following day, mice were injected with ketamine intraperitoneally using an insulin syringe, at the indicated dose, and placed immediately in the activity chamber.

#### *Atp1a3*^+/D801Y^ mice only

##### Wire traverse

For strength and coordination in D801Y heterozygotes, the elevated beam apparatus was modified by substituting a 3-mm-diameter, 36-inch-long metal rod, too narrow to walk on, for the wider wood rod. The mice cling upside down to the rod and move using all four feet and tail toward the support poles.

##### Postswim tremor

Tremor amplitude and frequency were measured via an iPhone accelerometer using an app for parkinsonism, LiftPulse, that is no longer available. The phone rested on four rubber pipette bulbs taped to a solid surface, and the mouse was placed on it within 1 min of a 15 min swimming session at 25°C. Ten consecutive tremor measurements per mouse were taken by restarting the app, which had a 15 s cycle time. After eliminating any measurement where the (WT) mouse walked off the phone, the measurements were averaged.

### Statistical analysis

Unless otherwise noted, data are reported as mean ± SD. Statistical analysis was carried out either with unpaired Student’s *t* test or with two-way ANOVA or mixed-effects model (RELM), followed by uncorrected Fisher's LSD test. The significance of litter survival proportions was determined by binomial test of observed to expected results with Fisher's exact test. A *p* value <0.05 was considered significant.

## Results

### Breeding, survival, and weight

Recent work with independent D801N and E815K mouse strains found evidence of mortality (Hunanyan et al., 2015; Helseth et al., 2018). Here, routine records were kept of birth, and genotyping was normally done at weaning. Table 1 shows differences in the impact of each mutation. Survival of pups at weaning age was similar in *Atp1a*3^tm1Ling/+^ and *Atp1a3*^+/D801Y^ mice at 98 and 94%. Breeding of heterozygotes with WT mice is expected to produce 50% heterozygous offspring; however, when genotyped at weaning, the expected 50:50 distribution was obtained only with the heterozygous *Atp1a*3^tm1Ling/+^ line. There were 32% fewer heterozygotes than wild type in the *Atp1a3*^+/D801Y^ strain compared with almost 50% fewer on the C57BL/6J (Janvier) background in the initial report (Holm et al., 2016).

**Table 1.** Colony breeding and survival in the three *Atp1a3* het strains

	*Atp1a*3^tm1Ling/+^		D801Y		D801N			
Litter size and survival to weaning								
Strain	6N		6N		6J		6J	
Litters w pup counts	66		66		14 on P0		32 at wean	
Pups/litter born	7.24		7.05		6.2		n.d.	
Pups/litter weaned	7.03		6.61		n.d.		6.6	
Survival	98.0%		93.8%		n.d.		(reduced)	
Proportion of mutants relative to wild type								
Litters genotyped	105 litters at wean		82 litters at wean		>50 litters on P0^a^		32 at wean	
Genotype	WT	Het	WT	Het	WT	Het	WT	Het
Number of pups	384	369	282	192	219	145	152	59
Proportion	0.51	0.49	0.595	0.405	0.602	0.398	0.72	0.28
Fisher's exact test		*p* = 0.757		*p* = 0.0041		*p* = 0.0073		*p* ≤ 0.0001
Hets as % of WT		96%		68%		66%		39%
Binomial test		*p* = 0.610		*p* ≤ 0.0001				*p* ≤ 0.0001

The genetic strain was C57BL/6N for *Atp1a*3^tm1Ling/+^ and *Atp1a3*^+/D801Y^ mice and C57BL/6J for *Atp1a3*^+/D801N^. Litter size and survival to weaning was calculated for *Atp1a*3^tm1Ling/+^, D801Y, and D801N. Calculated proportions of surviving heterozygotes and WT at weaning, as well as at P0–P1 (D801N only) showed a non-Mendelian yield. The significance of WT and heterozygote proportions was determined by binomial test of observed to expected results with Fisher's exact test. Chi squared analyses gave qualitatively the same result but are regarded as approximations rather than exact tests. Runts and litters that failed due to lack of maternal care were not included because this is not uncommon in inexperienced C57BL/6 dams.

a The experimental aims for these cohorts were to use females, and so most male pups were not genotyped on P0. The data shown here for P0 were ∼80% female pups.

This contrasts with the JAX *Atp1a3*^+/D801N^ strain where there were 61% fewer heterozygotes than WT at weaning. A separate, smaller cohort of *Atp1a3*^+/D801N^ mice was genotyped at birth. These showed 19% fewer heterozygotes than wild types, but without statistical significance (Table 1). To our knowledge survival records have not been published for any of the other *Atp1a3* mouse strains. The cause of pre- or perinatal death is not known, but it is not due to maternal mutation because dams were routinely WT.

**Figure 1.**
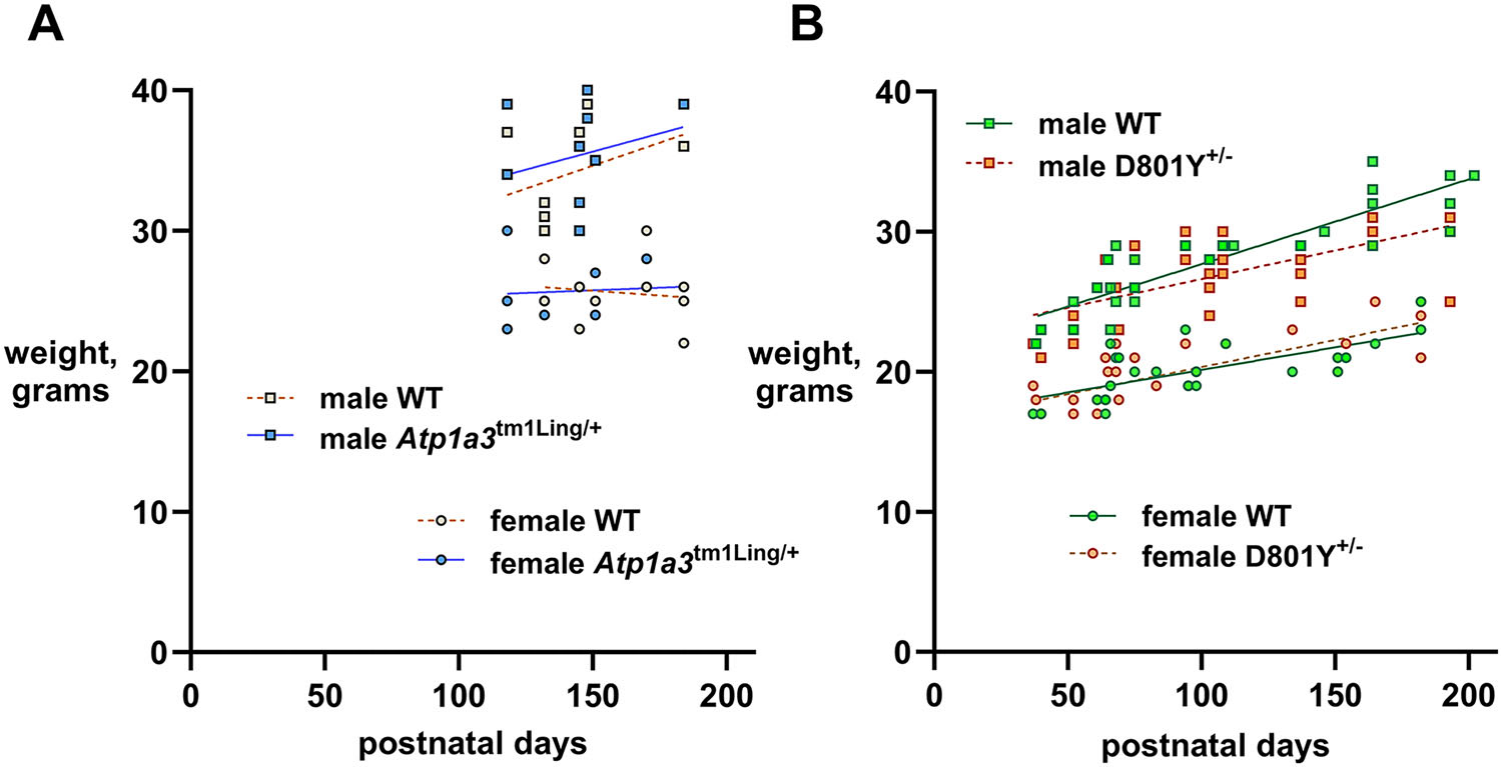
Body weights of *Atp1a*3^tm1Ling/+^ and *Atp1a3*^+/D801Y^. ***A***, Weights of *Atp1a*3^tm1Ling/+^ mice were recorded at the time of motor testing, from 4 to 6 months of age. No differences were seen in males between WT (*n* = 8) and heterozygotes (het; *n* = 13) or females (*n* = 10 for both WT and het). Solid lines represent linear regressions for wild type. Dashed lines represent linear regressions for *Atp1a*3^tm1Ling/+^, not significant by analysis of covariance (ANCOVA). ***B***, Weights of *Atp1a3*^+/D801Y^ mice from 5 weeks to 6 months of age. Green symbols indicate wild type, ochre symbols indicate *Atp1a3*^+/D801Y^. Solid lines represent linear regressions for wild type. Dashed lines represent linear regressions for *Atp1a3*^+/D801Y^. Analysis of covariance (ANCOVA) was used to compare regression lines between WT and *Atp1a3*^+/D801Y^. While slopes were not different in females (WT = 0.004605; *Atp1a3*^+/D801Y ^= 0.009857; *F* = 0.1559; DFn = 1, DFd = 50; *p *= 0.06946), a statistically significant difference (*p *= 0.0356) was observed between WT and *Atp1a3*^+/D801Y^ slopes in males (0.06053 and 0.04080, respectively; *F* = 4.585; DFn = 1, DFd = 72).

Reduction of average weight by 11% was reported previously in the first, independently produced D801N mutant mouse line (Hunanyan et al., 2015). Here, weights for the *Atp1a*3^tm1Ling/+^ and *Atp1a3*^+/D801Y^ lines were recorded at the time of experimental testing. Females did not differ from WT in either line (Fig. 1*A*,*B*). *Atp1a3*^+/D801Y^ males showed a trend to lower weights only above 4 months of age; linear regression analysis found a significant difference (*p *= 0.036; Fig. 1*B*).

### Comparing α3 isoform expression and Na,K-ATPase activity

#### Expression

**Figure 2.**
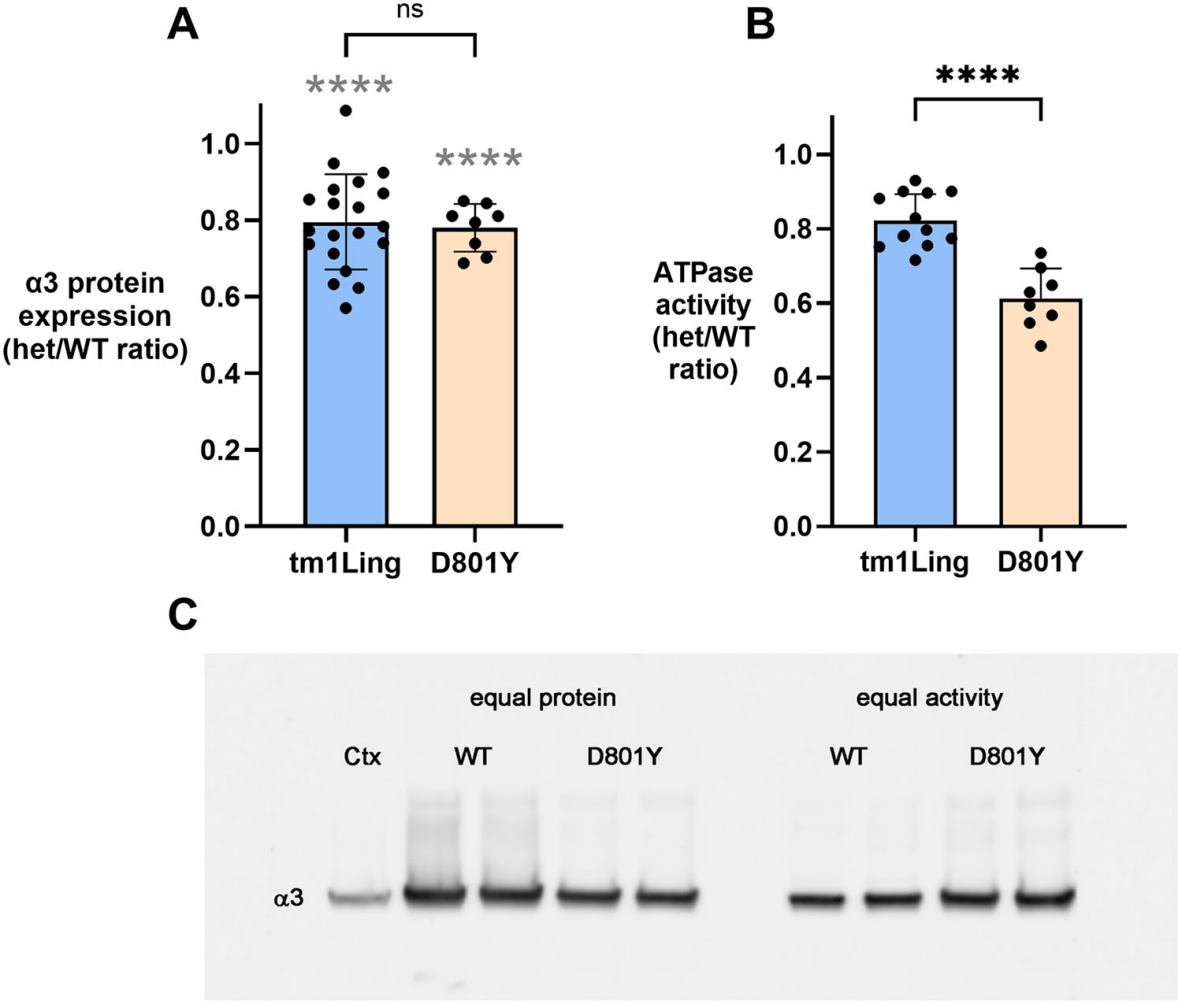
Expression levels of α3 and ATPase activity in *Atp1a*3^tm1Ling/+^ and *Atp1a3*^+/D801Y^ mice. ***A***, Relative expression level of the α3 subunit was determined by Western blots of crude homogenates. Each datapoint is a within-experiment ratio of observed antibody stain for mutant relative to WT for *Atp1a*3^tm1Ling/+^ (tm1Ling) and *Atp1a3*^+/D801Y^ (D801Y) mice. Statistical analysis was performed with Student's *t* test. *p* values are as follows. For the ratio of each mutant to WT, *p* < 0.0001 (gray asterisks); for comparison between mutant strains: protein expression *p = *0.18, coincidentally the same. ***B***, The maximal ouabain-sensitive ATP hydrolysis was similarly measured in preparations from *Atp1a*3^tm1Ling/+^ and *Atp1a3*^+/D801Y^ mice. For reduction of ATPase activity relative to WT, *p* ≤ 0.0001 for both strains. In comparing the strains, the greater reduction in *Atp1a3*^+/D801Y^ was also *p* ≤ 0.0001. ***C***, α3 subunit Western blots were also carried out loading equal amounts of protein (left) or equal amounts of activity (right). The protein concentrations and ATPase activities for each preparation were determined together. We then prepared a single 80 µl of complete LDS (SDS) sample with equal amounts of protein for each preparation. On the equal protein side of the gel, 20 µl of these were loaded into gel wells. On the equal ATPase activity side, a calculated volume of each master sample was added corresponding to equal amounts of measured ATPase activity. The samples on both sides of the gel thus came from the same tubes.

Fundamental expectations in mutant mice are that a null mutation should result in less protein (at most a loss of 50%) and that missense mutations may result in less protein if the mutation reduces biosynthesis. Differences in expression of the α3 isoform were determined by Western blot. Figure 2*A* shows two different things, the ratio of mutant activity to WT activity for each strain, each of which was significantly lower than its littermate controls (*p* < 0.001). Then it shows that the amounts of α3 protein in the *Atp1a*3^tm1Ling/+^ and *Atp1a3*^+/D801Y^ mice were coincidentally not different from each other (n.s. by *t* test). Notably, the *Atp1a*3^tm1Ling/+^ mice exhibited much higher α3 protein levels than the theoretical 50% level: an average of 82% (*p *< 0.0001 vs littermate WT controls, *n* = 20; Fig. 2*A*). This observation is not consistent with the original report of the same strain on a Black Swiss background (Moseley et al., 2007) where a 60% reduction of total α3 protein was reported. Na,K-ATPase α3 subunit expression level was not measured in three other studies with the same mice (Clapcote et al., 2009; DeAndrade et al., 2011; Kirshenbaum et al., 2011b) or in an independent knock-out strain (Ikeda et al., 2013). The recovery or >50% implies either that the amount of mRNA produced is not normally the limiting factor or that in this particular case there is production of α3 protein with a Ser-Val insertion at the splice site due to the use of an alternative splice donor two codons downstream (see explanation in Materials and Methods, Animals).

The *Atp1a3*^+/D801Y^ mice also exhibited significantly less α3 protein than 100% of the WT level (average 78%; *p *< 0.0001; *n* = 8; Fig. 2*A*), confirming its original report (Holm et al., 2016). If we assume that 50% of the total is from the wild-type allele and the other 28% from the mutant allele, this implies a 44% reduction of the mutant protein. This is presumably due either to reduced biosynthesis or more rapid degradation of the mutant protein. Reduced expression of the α3 isoform harboring the D801Y mutation was also seen in transiently transfected cells (de Carvalho Aguiar et al., 2004; Heinzen et al., 2012).

#### ATPase activity

In principle, either a null mutation or a missense mutation could result in a 50% loss of enzyme activity, but this is by no means certain. In mice (but not in humans), the affinity of the α1 isoform for the specific inhibitor ouabain is 100-fold reduced compared with α2 and α3 and that makes it possible to assay α1 separately from α2 + α3. In adult mice and rats, α2 is virtually limited to glia and makes a small contribution to the total (estimated to be 10%), while the α1 isoform, which is expressed in most neurons, is ∼20% under *V*_max_ conditions. Maximal ATP hydrolysis sensitive to 10 μM ouabain was assessed in preparations from *Atp1a*3^tm1Ling/+^ and *Atp1a3*^+/D801Y^ mice (Fig. 2*B*). In agreement with a previous finding (Kirshenbaum et al., 2011b), in the *Atp1a*3^tm1Ling/+^ mice the level of activity was 80%, corresponding here to the level of α3 protein. It is not clear whether the extra 30% activity represents active enzyme from an aberrant transcript with a two amino acid insertion or whether the level of α subunit from the WT allele exceeds 50% because it is limited by a post-transcriptional step and not by its mRNA. Figure 2*B* shows that in *Atp1a3*^+/D801Y^ mice, there was a larger reduction of activity relative to WT, but not to 50%. It has been shown in *Xenopus* oocytes that the D801Y mutation in *Xenopus* Atp1a1 abolishes pump-mediated activity at room temperature (Isaksen et al., 2017) and that cells with the same mutation in HEK293T at 37°C do not survive the inhibition of endogenous α1 by ouabain (de Carvalho Aguiar et al., 2004). Given that a normal amount of activity is expected to come from the wild-type allele, we estimate that the specific activity of enzyme from the mutant D801Y allele is impaired by at least 75%, consistent with measurements made with transiently transfected cells (Heinzen et al., 2012). This is likely an underestimate because of the presence of α2 from glia. There was also comparably less activity when normalized to the units of α3 protein detected on Western blotting after loading either equal amounts of total protein (Fig. 2*C*, left) or an amount of protein calculated to correspond to equal activity based on measured activity per microgram of protein (Fig. 2*C*, right). This was done by preparing a single oversized SDS sample for each preparation and loading volumes corresponding to either equal protein or equal Na,K-ATPase activity. Densitometry showed a 27% lower level of total α3 protein in the D801Y mutant, resulting in a corresponding greater loading of α3 protein when activity is equalized.

Altogether, Figure 2 shows that the *Atp1a*3^tm1Ling/+^ mouse and the D801Y knock-in brain samples have coincidentally similar levels of α3 protein but that the knock-in has considerably less Na,K-ATPase activity. The difference in activity is compatible with the behavioral differences that were measured here and in prior work cited above, without ruling out gain of toxic function effects. Another possibility is the existence of a dominant-negative effect for the D801Y mutation, which may explain the observed differences in α3 activity despite similar protein expression, as already established for the human AHC-causing mutations D801N, G947R, and E815K (Li et al., 2015). A reduction of the WT protein can also occur when there is a major perturbation of protein biosynthesis caused by a severe mutation. An example is the reduction of the biosynthesis of endogenous WT α1 along with mutant in cells expressing ATP1A3 L924P (Arystarkhova et al., 2021).

### Phenotypes of the *Atp1a*3^tm1Ling/+^ mice

#### Motor testing

Because most *ATP1A3* mutations in humans produce motor symptoms, and because motor skill testing in mice is one preferred way to document and quantify phenotype, motor impairment was assessed.

In the home cage and during handling, heterozygote *Atp1a*3^tm1Ling/+^ mice could not be distinguished from their WT littermates. Motor screening was done with age- and sex-matched mice with six per group. When suspended by the tail, heterozygotes showed typical posture with hindlimbs splayed. An observational SHIRPA screen did not detect any gross abnormalities. Other tests of supraspinal motor control that detected no deficits were the sticker swipe test, which measures speed to use the forelimbs to remove a sticker placed on the head, and the ability to walk across crumpled chicken wire.

**Figure 3.**
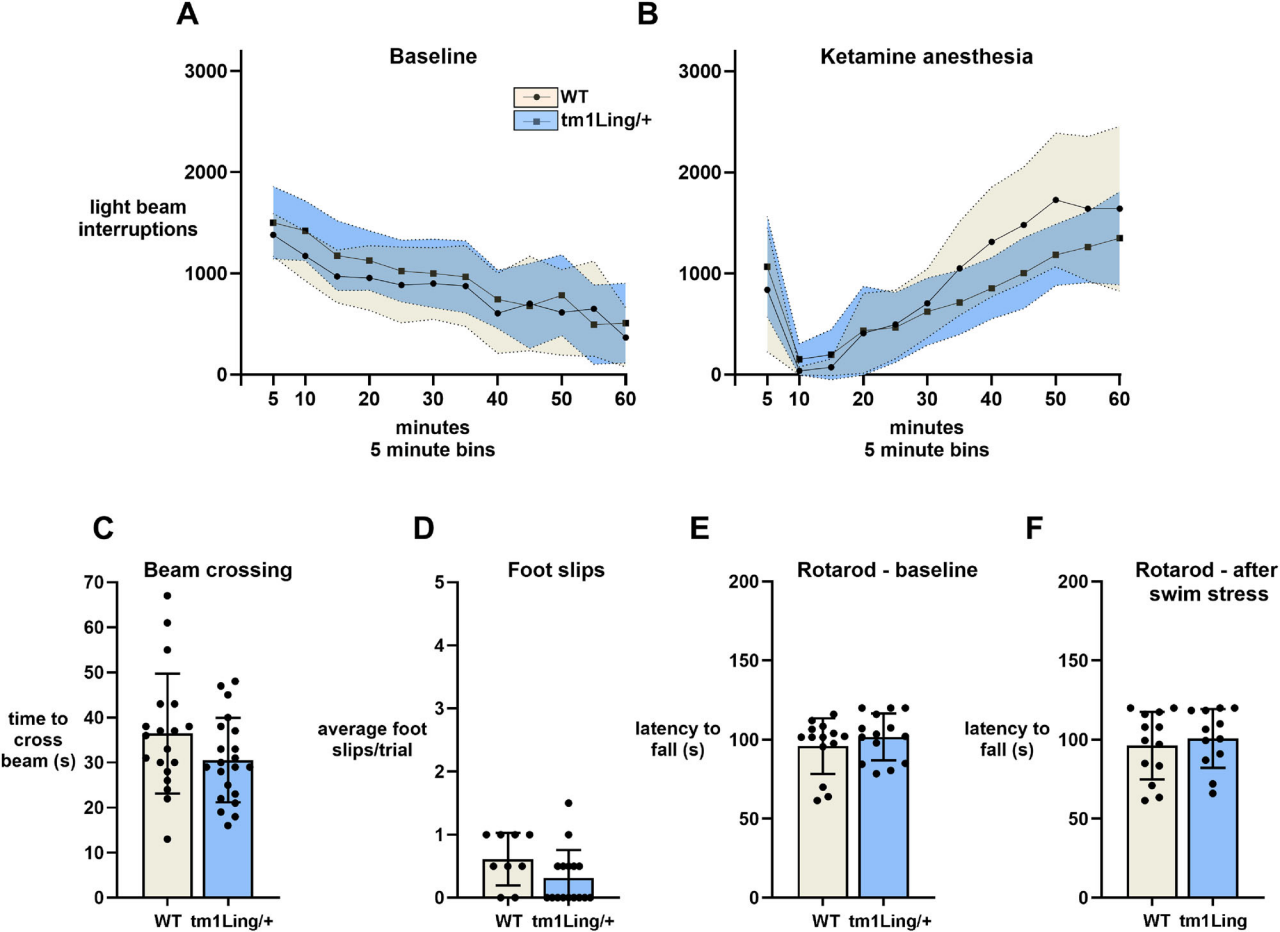
Activity and motor function of *Atp1a*3^tm1Ling/+^ mice. Baseline open-field chamber activity was evaluated in WT (*n* = 6) and *Atp1a*3^tm1Ling/+^ (*n* = 6), by quantifying optical beam breaks during a 1 h test (***A***). No difference in activity was observed between WT and *Atp1a*3^tm1Ling/+^. Data are shown as mean ± SD (shaded areas). Statistical analysis was performed by two-way ANOVA, followed by uncorrected Fisher's LSD test. The same activity protocol was repeated after administration of 100 mg/kg ketamine (***B***). No significant difference was recorded either in the duration of full immobility at ∼5 min or in the rate of recovery. Motor coordination and balance was quantified by measuring the time necessary to cross the beam (***C***) and the number of paw slips (***D***) that occur in the process. The accelerating rod also did not show difference between *Atp1a*3^tm1Ling/+^ and WT under baseline conditions (***E***) or 1 month after stress elicited by forced swimming for 15 min every day for 5 d (***F***).

Hyperactivity in an open-field environment has been observed in most *Atp1a3* mutant mice (summarized in Table 3). In the open-field chamber, however, *Atp1a*3^tm1Ling/+^ mice were not significantly different from WT. Activity was monitored for 60 min. When the number of beam breaks was plotted versus time and tested in a linear regression analysis (Fig. 3*A*, dashed lines), slopes did not significantly differ between the two groups (−14.34, −17.54, respectively, for WT and het; *p *= 0.136).

The same activity protocol was used to assess the response to a minimally anesthetic dose of ketamine, 100 mg/kg (Fig. 3*B*). The relevance of the normal response of *Atp1a*3^tm1Ling/+^ mice to the drug will become clear when compared with Figure 6 with the *Atp1a3*^+/D801Y^ mouse. In *Atp1a*3^tm1Ling/+^ and WT controls, the length of full immobility was not significantly different, both ∼5 min. The rate of recovery was also not significantly different.

The elevated beam test is used to assess motor coordination and balance in mildly stressful conditions. The mouse is forced to walk across a narrow beam to a safe platform. Performance on the beam is quantified by measuring the time necessary to cross the beam (Fig. 3*C*) and the number of paw slips that occur (Fig. 3*D*). Symptoms on the beam were absent in *Atp1a*3^tm1Ling/+^ heterozygotes of either sex.

The accelerating rotarod is routinely used to detect motor deficits in mutant mice. Here we observed no difference between *Atp1a*3^tm1Ling/+^ mice and WT (Fig. 3*E*). This is similar to prior work: a transient superior performance was reported for the Kawakami *Atp1a3* heterozygote knock-out (Ikeda et al., 2013), and no effect was reported for the Lingrel *Atp1a*3^tm1Ling/+^ line at baseline (DeAndrade et al., 2011).

**Figure 4.**
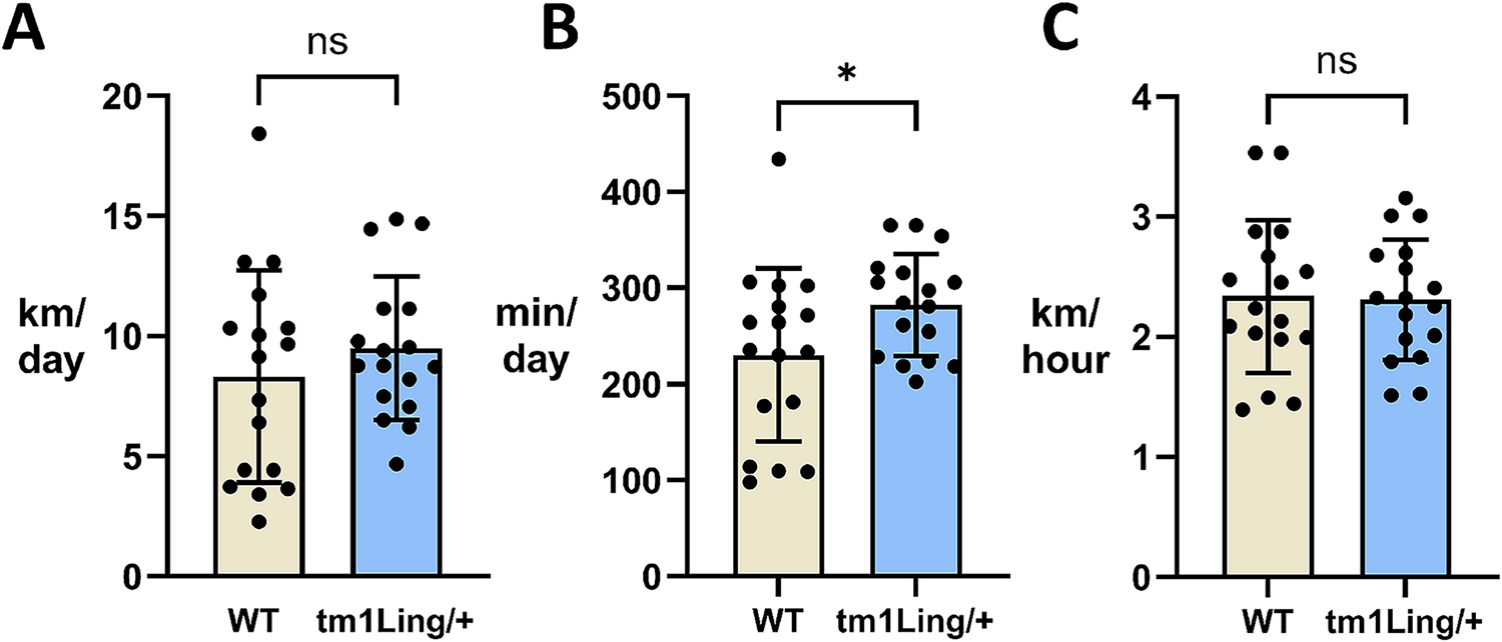
*Atp1a*3^tm1Ling/+^ mice exhibit normal running parameters. Running on metered wheels was used to look for deficits in the *Atp1a*3^tm1Ling/+^. Data were collected for 9 consecutive days (*n* = 16 mice per group). There was no effect of genotype on two parameters, kilometer run per day (***A***), and speed in km/hour (***C***). Time (min) spent running per day (***B***) showed a statistically significant difference between the two groups (*p *= 0.0472). Data are shown as mean ± SEM.

Human mutations in *ATP1A3* were first discovered because of RDP which is often triggered by stressful events. In the literature on *Atp1a3* mice, application of stress sometimes elicits symptoms (Kirshenbaum et al., 2011b; Sugimoto et al., 2014; Ng et al., 2021). Consequently, the same cohort of mice were then stressed by forced swimming for 15 min every day for 5 d (Fig. 3*F*). Rotarod performance was unaffected. When given running wheels in their home cages, *Atp1a*3^tm1Ling/+^ mice ran essentially normal running times and distances (Fig. 4*A–C*).

#### Repeated ethanol stress

**Figure 5.**
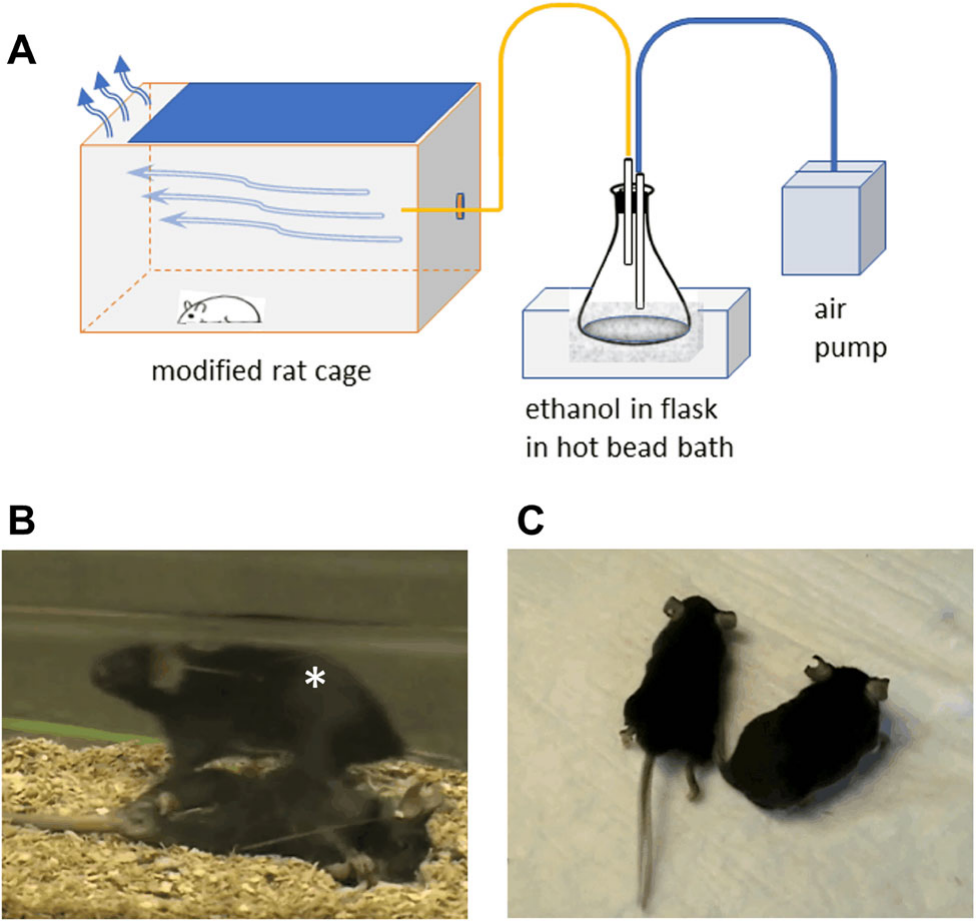
Ethanol stress reveals neurological vulnerability in *Atp1a*3^tm1Ling/+^ mice. WT and *Atp1a*3^tm1Ling/+^ mice were held in a vapor chamber (layout in ***A***) and exposed for 6 h every weekday for 2 weeks. ***B***, ***C***, The mutants are either unrecovered (***B***) or experiencing repetitive dystonic or myoclonic movements (***C***). The wild type in ***B*** is labeled with an asterisk. See also Movie 1.

Because moderate stress (forced swimming) did not result in symptoms, we opted to try to mimic binge drinking in *Atp1a*3^tm1Ling/+^ mice, something that can trigger onset of persistent dystonia in RDP (Barbano et al., 2012). To obtain intoxication severe enough to cause loss of consciousness, mice were held in a vapor chamber (Fig. 5*A*) and exposed for 6 h every weekday for 2 weeks. In pilot experiments, mice were treated in groups and free to interact, and running wheels allowed us to observe a phase of hyperactivity. With more advanced intoxication, however, the mice piled up in a corner. To monitor them better, they were individually confined to upside-down metal mesh pencil cups within the chamber, each with a container of gel diet for nourishment and hydration. After recovery, none of the mice exhibited noticeable seizures, even when challenged by gentle spinning when suspended by the tail. Unlike WT mice, however, after multiple days of treatment, four of eight stressed *Atp1a*3^tm1Ling/+^ mice exhibited extended hindlimbs and hyperkinetic motor abnormalities (Fig. 5*B*,*C*). Movie 1 shows several examples of littermate wild types and heterozygotes at the same time point during recovery from ethanol exposure. Each impaired *Atp1a*3^tm1Ling/+^ mouse repeated the same unique abnormality on subsequent days of ethanol treatment. None of the WT mice exhibited behaviors other than hyperactivity, stupor, and loss of consciousness, and they recovered faster. This is evidence that even a 20% reduction of α3 protein and ATPase activity affects the neurological vulnerability of the mouse.

### Phenotypes of the knock-in *Atp1a3*^+/D801Y^ mice

#### Posture and open-field activity

Adult *Atp1a3*^+/D801Y^ mice showed differences in posture when WT and *Atp1a3*^+/D801Y^ littermates were compared (Movie 2). Experienced observers could identify them at 2–3 months of age when blinded to genotype. They appeared flatter, suggesting lower muscle tone, and in some instances displayed a mild kyphosis.

**Figure 6.**
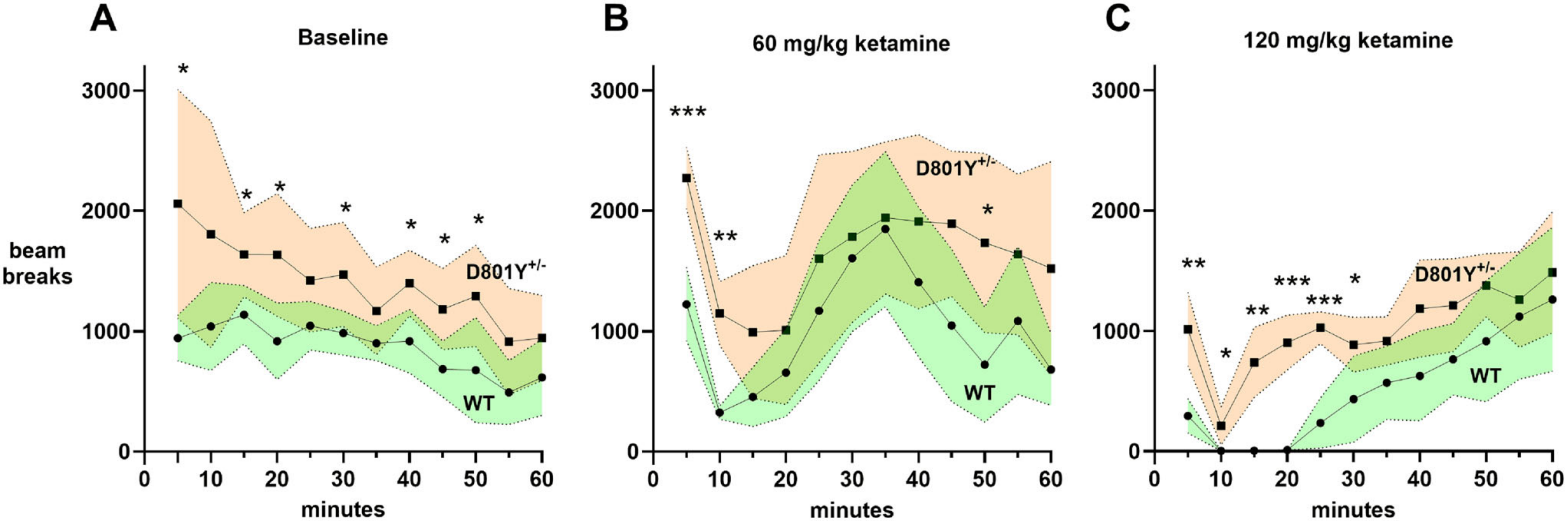
*Atp1a3*^+/D801Y^ hyperactivity and paradoxical response to ketamine. Open-field activity was assessed for 60 min in WT and *Atp1a3*^+/D801Y^ mice at baseline (***A***; *n* = 6 per group) and upon administration of 60 mg/kg (***B***) and 120 mg/kg (***C***) ketamine (*n* = 5 per group for both doses). *Atp1a3*^+/D801Y^ mice displayed a hyperactive behavior relative to WT at most of the time points tested (*p* < 0.0001). A subanesthetic dose (***B***) produced robust rebound-associated hyperactivity. At high anesthetic dose (***C***), *Atp1a3*^+/D801Y^ mice never lost consciousness and exhibited dyskinesias. See also Movie 3. Data are shown as mean ± SD (shaded areas). Statistical analysis was performed by two-way ANOVA (or RELM mixed-model for data relative to 120 mg/kg dose), followed by uncorrected Fisher's LSD post hoc test. Asterisks denote statistically significant differences between groups at different time points. Individual time point *t* tests that were significant had *p* values as follows: baseline: at 5’ = 0.0341; at 15 min = 0.0192; at 20 min = 0.0182; at 30 min = 0.0395; at 40 min = 0.0108; at 45 min = 0.0155; at 50 min = 0.0322. 60 mg/kg ketamine: at 5’ = 0.0004; at 10’ = 0.0018; at 50’ = 0.0385. 120 mg/kg ketamine: at 5’ = 0.0036; at 10’ = 0.0426; at 15’ = 0.0050; at 20’ = 0.0009; at 25’ = 0.0002; at 30’ = 0.0499.

In agreement with previous findings (Holm et al., 2016), *Atp1a3*^+/D801Y^ mice were more active than WT in the open-field test. For this strain, there was a profoundly significant difference between linear regression slopes of WT and *Atp1a3*^+/D801Y^ (−9.383, −17.70, respectively; *p *< 0.0001; Fig. 6*A*).

#### Paradoxical response to anesthesia in *Atp1a3*^+/D801Y^ mice

Although we normally used isoflurane for routine anesthesia, a pilot experiment using ketamine–xylazine was halted because of the *Atp1a3*^+/D801Y^ mouse's abnormal response. Ketamine is a quick-acting general anesthetic and is a glutamate receptor *N*-methyl-d-aspartate (NMDA) antagonist used for induction of anesthesia in diagnostic and surgical procedures. A subanesthetic dose of ketamine alone (60 mg/kg) sedated the WT mice, followed by a marked rebound in open-field activity (Fig. 6*B*). The same dose in *Atp1a3*^+/D801Y^ mice had much less sedative effect but was also followed by a rebound in activity. At this lower ketamine dose, they were more active than WT at 5 min (by 2-fold), 10 min (3.5-fold), and 50 min (2.4-fold). In pilot experiments, 100 mg/kg (the dose used for *Atp1a*3^tm1Ling/+^ experiments shown in Fig. 3) did not anesthetize *Atp1a3*^+/D801Y^ mice, and so the dose was increased for further testing. Administration of 120 mg/kg produced an appropriate response in WT animals, with complete anesthesia recorded between 10 and 20 min, whereas it failed to completely anesthetize *Atp1a3*^+/D801Y^ mice (Fig. 6*C*). Activity was also increased, at 5 min (3.4-fold), 15 min (123-fold), 20 min (78-fold), and 25 min (4.4-fold) at the higher dose (Fig. 6*C*). Movie 3 shows the paradoxical response of *Atp1a3*^+/D801Y^ mice to 120 mg/kg ketamine at 3, 8, and 12 min. Some behaviors resembled tonic–clonic seizures in motor output.

#### Motor tests

Unlike *Atp1a*3^tm1Ling/+^ mice, *Atp1a3*^+/D801Y^ mice had resting symptoms (low posture) and showed a phenotype in all of the motor tests applied.

**Figure 7.**
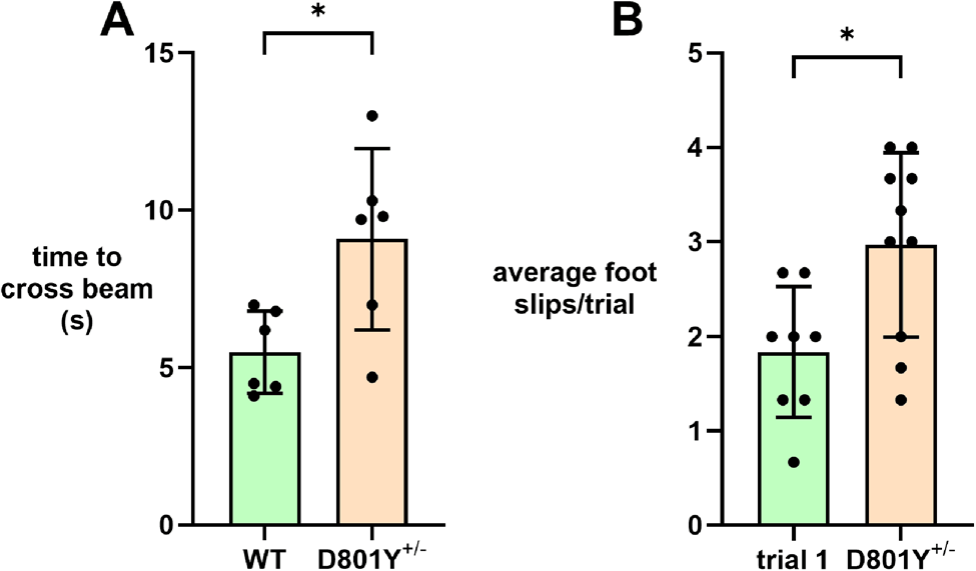
Beam cross and performance in *Atp1a3*^+/D801Y^. Motor coordination and balance were assessed by measuring performance on the balance beam. This test, with females only, was conducted a total of three times for each mouse and triplicates were averaged. Time to cross the beam (***A***) and number of paw slips (***B***) were quantified. Statistical analysis was performed by unpaired Student's *t* test. *p* values are as follows: *p *= 0.0195 and 0.0136 for time to cross beam and foot slip, respectively.

In the beam-crossing test, *Atp1a3*^+/D801Y^ mice had significantly more foot slips and were slower to cross than WT (Fig. 7*A*,*B*). Movie 4 shows differences in motor coordination and balance in *Atp1a3*^+/D801Y^ mice, which exhibit extremely dystonic posture and evident contralateral slipping on the beam.

An incidental behavioral observation was made during the beam test that we report here for its potential significance and utility. In one of the human *ATP1A3* syndromes (CAPOS, *ATP1A3* p.Glu818Lys), a loss of retinal ganglion cells occurs in patients as detected by optic nerve atrophy and loss of vision (Demos et al., 2014). Retinal photoreceptors, ganglion cells, and the optic nerve originating in retinal ganglion cells express an exceptionally high level of α3 protein (Wetzel et al., 1999). In unrelated experiments, we had observed that blind mice (the FVB strain, homozygous for the recessive rd1 allele of the gene *Pde6b*) placed on the beam will reach far down to try to touch a surface with their whiskers, while C57BL/6NCrl and sighted F1 hybrids of FVB and C57BL/6NCrl do not. Here the behavior of both *Atp1a*3^tm1Ling/+^ and *Atp1a3*^+/D801Y^ strains was consistent with normal vision on the elevated beam. Furthermore, retinal ganglion cells were examined and counted in a flat retinal preparation from wild-type and *Atp1a3*^+/D801Y^ mice, and their numbers per unit area were found to be normal in all regions sampled (*n* = 4 per group; *p *= 0.3; Table 2).

**Table 2.** Ganglion cell counts in *Atp1a3*^+/D801Y^ mouse retina

Cells/mm^2^	Retinal region	Inferior	Nasal	Superior	Temporal	Average
WT 1	Left	4,440	2,830	3,812	4,783	
WT 2		4,712	4,535	3,291	3,587	
WT 3		4,973	4,949	3,138	4,594	
WT 4		4,428	4,700	4,771	2,510	
WT1	Right	4,985	4,641	3,398	4,641	
WT2		4,689	4,677	4,996	4,073	
WT3		4,677	5,221	5,304	4,156	
WT4		4,487	4,712	2,913	4,061	4,303 ± 738 SD
*Atp1a3* ^+/D801Y^						*p *= 0.296
het 1	Left	4,914	4,322	1,954	3,209	
het 2		5,363	4,641	3,173	4,582	
het 3		3,291	5,233	2,640	2,984	
het 4		3,966	4,191	2,569	3,848	
het 1	Right	4,440	3,753	4,499	5,174	
het 2		5,435	4,949	5,340	3,244	
het 3		4,606	4,771	5,363	2,889	
het 4		4,381	4,274	3,694	2,735	4,076 ± 968 SD

Whole mounts of the isolated retinas, with regular cuts for flattening, were stained for β3 tubulin as a ganglion cell marker. Images of the ganglion cell layer were collected with a confocal microscope at 40× in mid-periphery (i.e., halfway between the optic nerve head and the rim of the retina). Ganglion cells were counted in ImageJ.

**Figure 8.**
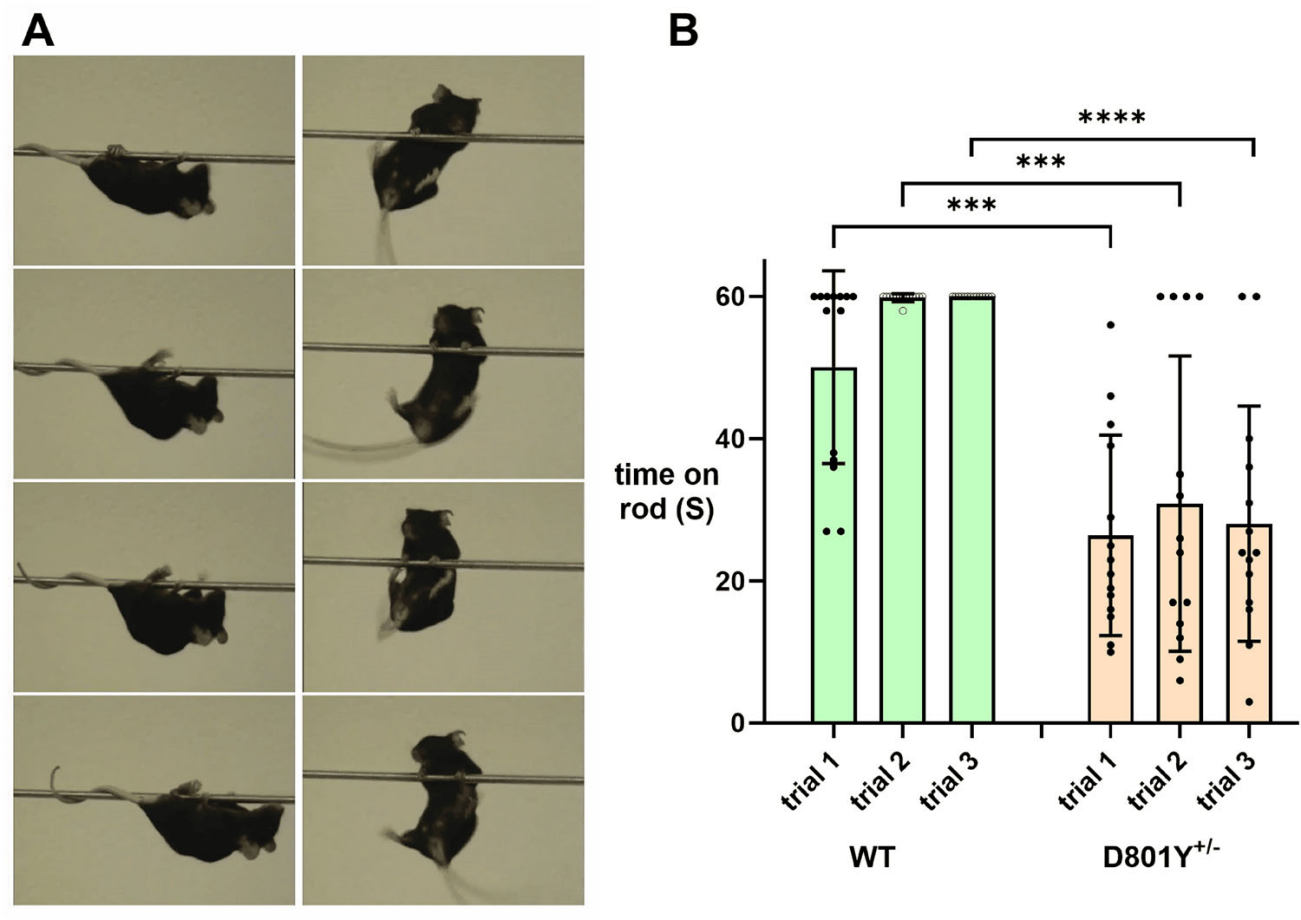
Weakness of the *Atp1a3*^+/D801Y^ heterozygotes on the narrow rod. A 3 mm rod suspended between two posts was a more stringent challenge than the beam. Each mouse was placed on the rod and given the opportunity to grasp it. Wild types clung to the rod and inched their way toward a post. *Atp1a3*^+/D801Y^ (D801Y) mice had great difficulty with their hindlimbs and rarely stayed on for the full 60 s. ***A***, Sequential photographs; WT is on the left. ***B***, Quantification for a cohort with *n* = 14 per group. Data were analyzed with unpaired *t* test with Welch's correction for unequal variance (*p* ≤ 0.0001; <0.0002; and <0.0001, respectively, for trials 1, 2, and 3).

While the beam-crossing test showed significance in the differences between WT and *Atp1a3*^+/D801Y^ mice, the test's emphasis on speedy execution of running did not capture their visible functional differences very well. In tests of grip strength assessed by the ability to cling to an inverted wire cage top, there were qualitative differences between the two strains. Wild-type mice and *Atp1a3*^+/D801Y^ showed no significant difference in length of time holding on (males, *n* = 7 WT and 8 HET; females, *n* = 17 WT and 16 HET), but there were adaptive behaviors: WT usually freely climbed around upside down, while heterozygotes moved less and sometimes used back paws like hooks and held on with wrapped tails. For this reason, we developed a more challenging test to better illustrate the apparent deficits in muscle strength and ability (Fig. 8*A*). A narrow metal rod was used in the elevated beam apparatus, too small to walk on, although wild-type mice occasionally managed to climb up and perch like birds. Wild-type mice used their forelimbs to pull themselves upside down to the end of the rod, where they could descend the attached pole. *Atp1a3*^+/D801Y^ mice struggled to hang on with their forelimbs. They were not able to raise their hindlimbs, and they seldom stayed on for the full 60 s. Figure 8*B* shows that WT mice improved their performance after the first trial, but most *Atp1a3*^+/D801Y^ mice could not (*n* = 14 per group).

**Figure 9.**
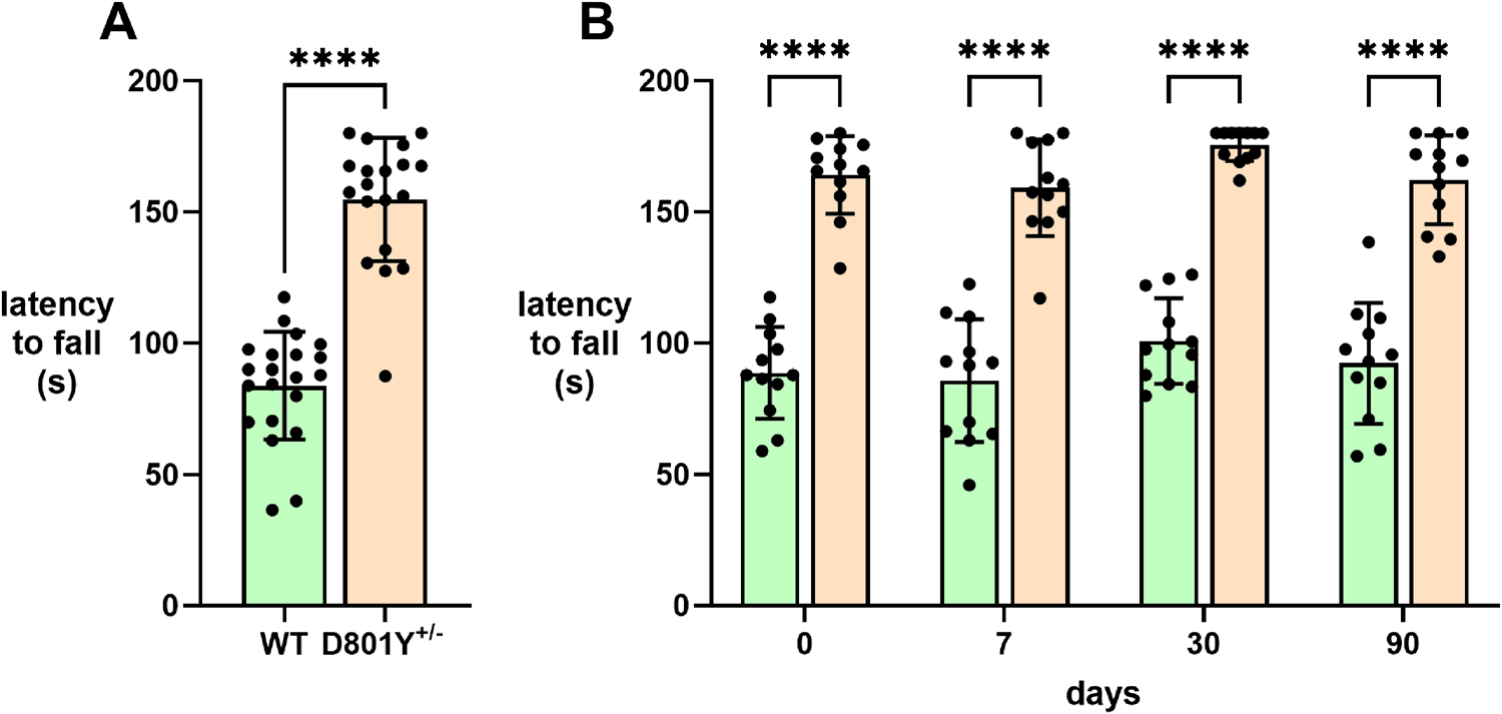
*Atp1a3*^+/D801Y^ mice exhibit exceptional performance on the rotarod. ***A***, The rotarod (average of two trials carried out 3 d apart) showed increased baseline activity in *Atp1a3*^+/D801Y^ (D801Y) mice compared with WT. The test was terminated at 180 s. ***B***, In a separate cohort, mice underwent testing again at baseline and then were stressed by forced swimming for 15 min. Then they were tested on the rotarod again at 7, 30, and 90 d later. Statistical analysis was performed by two-way ANOVA followed by Fisher's LSD test. Asterisks indicate statistically significant differences between WT and het (*p *< 0.0001 for all comparisons).

Unexpectedly, in rotarod performance *Atp1a3*^+/D801Y^ mice showed robust and consistent superiority over WT. No time-dependent effect in rotarod performance was identified at any of the tested time points in either sex. The *Atp1a3*^+/D801Y^ mice stayed on the accelerating rotarod twice as long as WT at baseline (Fig. 9*A*). Because application of stress sometimes elicits symptoms, we tested forced swimming as a stressor (primary effects described below). A different cohort of *Atp1a3*^+/D801Y^ mice was swim stressed immediately after baseline rotarod testing and then was tested after 1 week, 1 month, and 3 months (Fig. 9*B*). The better rotarod performance was sustained. Although the WT mice never succeeded in staying on for the full 180 s of acceleration from 4 to 40 rpm, half of the *Atp1a3*^+/D801Y^ heterozygotes were able to stay on to the end (Fig. 9*A*,*B*).

#### Hyperactivity during swimming and motor abnormalities during recovery

**Figure 10.**
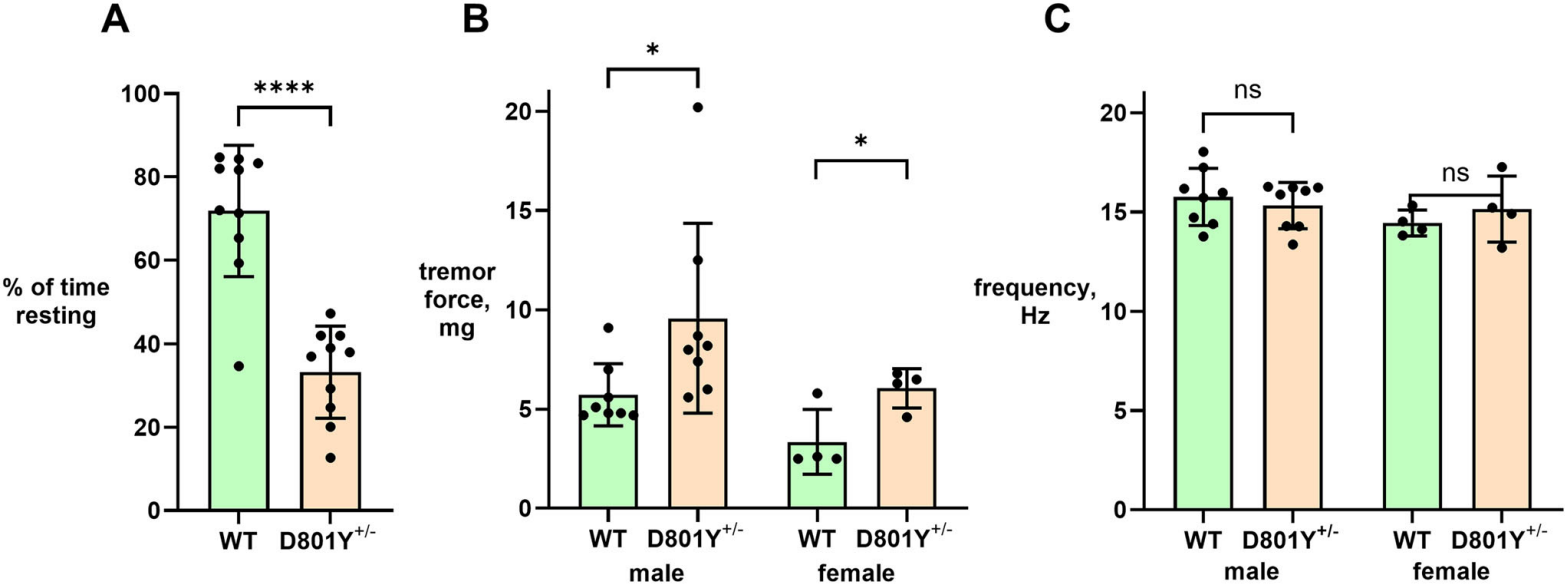
Hyperactivity of *Atp1a3*^+/D801Y^ in forced swimming; impairments during recovery. ***A***, C57BL/6 mice reach ∼70% immobility at 4–5 min, and so the time spent resting between 5 and 15 min was scored from video recordings. (The *Atp1a3*^+/D801Y^ mice that swam continuously were in unfilmed replicate experiments.) ***B***, ***C***, The tremor test detected an increase in amplitude over baseline in both males and females, but no significant difference in frequency.

When wild-type and *Atp1a*3^tm1Ling/+^ mice were exposed to forced swimming stress with the Porsolt protocol (total 15 min), they showed rapid swimming initially, but after several minutes, they would give up and float with no movements (or one-footed paddling) for a large part of the remaining time. This is typical: C57BL/6N mice reach ∼70% immobility at 4–5 min (Matsuo et al., 2010). In contrast, *Atp1a3*^+/D801Y^ mice in the same protocol showed persistent hyperactivity (Fig. 10*A*; Movie 5), often swimming continuously. Sometimes they appeared fatigued and one drowned; on two subsequent occasions, *Atp1a3*^+/D801Y^ mice were pulled out after 12–13 min to prevent drowning. All were monitored for 30 min during recovery. Wild types commenced grooming quickly, but *Atp1a3*^+/D801Y^ mice had a prolonged recovery with severe tremor and strikingly abnormal postures (Movie 6). Tremor was measured by accelerometer, and its force, but not its frequency, was changed (Fig. 10*C*,*D*). Of note, in the I810N mouse, swimming in tight circles was reported, and resting tremor amplitude and frequency were both elevated (Kirshenbaum et al., 2013). Swimming in cold water (16°C) produced similar postures and intense, spasmodic shivering (Movie 7), similar to the prior report where the same mice on the 6J background had their body temperatures reduced to 20°C by standing in cold water or by holding in a refrigerator (Isaksen et al., 2017).

**Movie 1.** Vulnerability of *Atp1a*3^tm1Ling/+^ mice to ethanol stress. Four of eight stressed *Atp1a*3^tm1Ling/+^ exhibited extended hindlimbs and hyperkinetic motor abnormalities during recovery from ethanol exposure. None of the WT mice exhibited abnormal behaviors (other than hyperactivity, stupor, and loss of consciousness) and had a faster recovery. Three sets of *Atp1a*3^tm1Ling/+^ and WT mice are shown in the video, followed by the fourth affected *Atp1a*3^tm1Ling/+^ alone. The asterisk indicates *Atp1a*3^tm1Ling/+^. [View online]

**Movie 2.** Lower posture in *Atp1a3*^+/D801Y^ heterozygotes. The video shows differences in posture of a WT and *Atp1a3*^+/D801Y^ (marked tail) littermates, when put in a novel environment. The *Atp1a3*^+/D801Y^ mouse appears lower, suggestive of lower muscle tone, and at times displays a mild kyphosis (abnormally curved spine). [View online]

**Movie 3.** Paradoxical response of *Atp1a3*^+/D801Y^ heterozygotes to ketamine anesthesia. A paradoxical response to 120 mg/kg ketamine was exhibited by *Atp1a3*^+/D801Y^ mice at 3, 8, and 12 min after injection. Unlike wild types, heterozygote mice were refractory to anesthesia at all time points even at this high ketamine dose; they never lost consciousness and they exhibited dyskinesias. [View online]

**Movie 4.** *Atp1a3*^+/D801Y^ mice performance on the balance beam. The *Atp1a3*^+/D801Y^ mice showed abnormal motor coordination and balance, consisting of extremely dystonic posture and evident slipping on the beam. [View online]

**Movie 5.** Persistent swimming in *Atp1a3*^+/D801Y^ mice. *Atp1a3*^+/D801Y^ mice and littermate wild types were filmed swimming in 25°C water for 15 min; *n* = 10/group. *Atp1a3*^+/D801Y^ mice show persistent hyperactivity, often swimming continuously. [View online]

**Movie 6.** Delayed recovery and tremor in *Atp1a3*^+/D801Y^ mice. The WT mouse recovering from the forced swim test is shown grooming, while the *Atp1a3*^+/D801Y^ mouse (marked tail) is immobilized by dystonia and a strong tremor, and later exhibited myoclonic jerks. [View online]

**Movie 7.** Persistent swimming in *Atp1a3*^+/D801Y^ mice at 16°C. *Atp1a3*^+/D801Y^ mice and littermate WT were filmed swimming in 16°C water. Similar to what was observed during swimming in room temperature water, at lower temperature *Atp1a3*^+/D801Y^ mice showed persistent hyperactivity, slow recovery, and spasmodic tremor. The asterisk indicates *Atp1a3*^+/D801Y^. [View online]

## Discussion

Symptomatic *Atp1a3* mutant mice are needed for insight into underlying pathogenic mechanisms and to be used for preclinical investigation of therapies. Perinatal survival was investigated in three *Atp1a3* mouse alleles, with differences that paralleled symptom severity. *Atp1a3*^+/D801N^ mice had the most mortality. Interestingly, prior work with an independent strain of D801N heterozygote mice showed cardiac abnormalities such as conduction delay when assessed under anesthesia (Balestrini et al., 2020). When seizures were induced with kainic acid, D801N mice in sinus rhythm developed conduction block or sinus arrest and died. This is published in a report of ATP1A3 patient resting ECG abnormalities, but because Atp1a3 is not detected in the mouse heart (O'Brien et al., 2000), Balestrini et al. proposed that the effect in their mice may instead be due to susceptibility to effects on the hypothalamic control of the autonomic system due to events such as spreading depolarization (Hunanyan et al., 2015) or to imbalance of excitation and inhibition (Hunanyan et al., 2018). The respiratory center in the brainstem is another potential source of mortality, because complete knock-out of Atp1a2 or Atp1a3 in mice blocked the generation of respiratory rhythm at birth (Moseley et al., 2003; Ikeda et al., 2017), and apnea occurs in a number of more severe ATP1A3 patients (Salles et al., 2021).

The behavioral data presented here add new insights into both the *Atp1a*3^tm1Ling/+^ and the *Atp1a3*^+/D801Y^ mice. We are cognizant that these two mouse models represent conditions rarely observed in patients with *ATP1A3* mutations, the former being a model for haploinsufficiency and the latter a mutation rarely observed in humans. The range of symptoms in humans with *ATP1A3* mutations is huge, from developmental brain malformation to late-onset dystonia, and mouse models also exhibit a wide range of phenotype manifestations. The study of these milder phenotypes is a useful approach to dissect the multifaceted nature of the *ATP1A3* mutation pathophysiology. Table 3 summarizes the most comparable motor test results in *Atp1a3* mouse models.

**Table 3.** Summary of comparable motor tests in *Atp1A3* mouse strains

	Reference	Activity	Beam	Rotarod	Grip	Swim	Strain
*Atp1a*3^tm1Ling/+^	Here	**=**					6N
	Moseley et al., 2007	**↑=** ^a^				**=↓** ^f^	Bl-Swiss
	DeAndrade et al., 2011		**=↓** ^c^	**=↓** ^c^	**=**		6N
*Atp1a3* ^+/−^	Ikeda et al., 2013	**↑**		**=↑** ^d^		**=** ^f^	6J
	Sugimoto et al., 2014		**=**	**=**		**=** ^f^	6J
	Sugimoto et al., 2018				**=**		
*Atp1a3* ^+/D801Y^	Here	**↑**	**↓**	**↑**	**↓** ^e^	**↑**	6N
	Holm et al., 2016	**↑**					6J
	Isaksen et al., 2017		**↓**				6J
*Atp1a3* ^+/I810N^	Kirshenbaum et al., 2011a	**↑**				**↑**	6N
	Kirshenbaum et al., 2013		**↓**	**=**	**=**	**↑**	6N
	Kirshenbaum et al., 2014	**↑**		**=**			6N
	Kirshenbaum et al., 2015					**↑** ^f^	6N
*Atp1a3* ^+/D801N^	Hunanyan et al., 2015	**↑**	**↓**	**=**	**=**	**↑** ^f^	6J
	Hunanyan et al., 2021			**=**	**=**		6J
	Uchitel et al., 2021			**=**	**=**		6J
*Atp1a3* ^+/E815K^	Helseth et al., 2018	**↓** ^b^	**↓**	**↓**	**=**	**?** ^g^	6J

This is not a comprehensive list of motor tests performed, but those most relevant for comparison here. An equals sign means no difference from WT. Up and down arrows indicate increases or decreases in performance relative to WT.

a Hyperactivity was seen only during the first 20 min.

b Hindlimb dragging.

c Only females were affected.

d There was unusually poor performance of WT given that they had 4 d of habituation.

e We saw qualitative differences in wire grip; see text and the more demanding rod hang test.

f Observations made during Morris water maze testing. The Morris water maze is used as a test of ability and memory under the implicit assumption that mice will not be abnormally fast. Published observations of swimming performance in this test in *Atp1a3* mice are the most nuanced because hyperactive or thigmotaxic swimming can result in longer times to reach the platform. An up arrow in most cases was assigned because of commentary consistent with such a possibility, but please read the source papers for detail.

g Special case: the water was 40°C, and mice had paroxysmal spells.

The present data on *Atp1a*3^tm1Ling/+^ mice contribute information pertinent to possible “loss of function” (LoF) alleles, defined as any genetic change that prevents the production of the protein and that might produce haploinsufficiency. There are more than a dozen LoF candidates in ClinVar and another 20 in gnomAD that may or may not be accompanied by loss of the protein. First, *Atp1a*3^tm1Ling/+^ mice had substantially more protein expression than 50%, matched by the level of activity. Nonetheless, unstressed heterozygotes have exhibited no disease-like symptoms when investigated in sufficiently powered experiments (Moseley et al., 2007; DeAndrade et al., 2011; Kirshenbaum et al., 2011b; Ikeda et al., 2013; Sugimoto et al., 2014). After restraint stress (DeAndrade et al., 2011; Sugimoto et al., 2014) and chronic variable stress (Kirshenbaum et al., 2011b), only some behavior indicators were altered. Here we detected neurologically incapacitating responses to chronic alcohol exposure, a severe stress intended to mimic the binge drinking that can be a trigger in some RDP patients. Curiously, there are case reports of onset of parkinsonian symptoms after binge drinking that exhibited bilateral lesions of the globus pallidus (Kuoppamaki et al., 2005; Del Brutto et al., 2022), a structure that was severely affected in postmortem pathology of RDP patients (Oblak et al., 2014). The true impact of loss of one *ATP1A3* allele is still unknown, but our findings on the *Atp1a*3^tm1Ling/+^ mice predict it would be mild.

D801 is a Na^+^ and K^+^-coordinating residue in the Na,K-ATPase ion binding site. There was a modest reduction in protein expression in *Atp1a3*^+/D801Y^ mice here that may be related to misfolding during biosynthesis (Arystarkhova et al., 2019), and not surprisingly, there was a greater reduction in ATPase activity than in *Atp1a*3^tm1Ling/+^ mice. The tests of the *Atp1a3*^+/D801Y^ mice contribute new facets of their phenotypes and confirm that their symptoms are less severe than in *Atp1a3*^+/D801N^ mice. D801N is the most common missense *ATP1A3* mutation in patients and is virtually always associated with classical AHC. In contrast, there are just three reports of D801Y in patients, presenting either with RDP (Ozelius, 2004; K. L. Wang et al., 2023) or mild AHC (Viollet et al., 2015). Both mutations substitute the negatively charged aspartate side chain with an uncharged but polar one, and why one has more severe consequences than the other is not fully understood.

Nevertheless, for the *Atp1a3*^+/D801Y^ mice, the present work confirms that there is some early (possibly prenatal) mortality during breeding, as well as increased activity in the open field (Holm et al., 2016). Here, we found that *Atp1a3*^+/D801Y^ adult mice had a deficit in posture or tone. Our observation of qualitatively different behaviors in the wire grip test led to a new hanging test on a very narrow beam that clearly showed weakness in the *Atp1a3*^+/D801Y^ mice compared with WT controls.

Another striking observation was that the *Atp1a3*^+/D801Y^ mice had hyperkinetic behavior during recovery from a subanesthetic dose of ketamine. Even a 20% higher than normal dose per kilogram body weight was unable to completely anesthetize them, and they showed spasmodic movements at peak ketamine effect. We speculate that the blockade of NMDA receptors on inhibitory neurons exacerbates an underlying hyperexcitability due to reduction of α3 ATPase activity, which results in a depolarizing shift in membrane potential as seen in mutant iPSC neurons (Simmons et al., 2018). This is highly relevant because AHC patients are reported to have an elevated rate of complications during sedation and anesthesia (Parker et al., 2022). Furthermore, physical and functional ATP1A3–NMDA receptor interactions have been reported (Akkuratov et al., 2015, 2020), highlighting the pump's physiological importance at the synapse. Holm et al. (2016) also rescued cognitive deficits with clonazepam, suggesting a therapeutic benefit from increasing levels of inhibition.

An even more surprising observation was that the *Atp1a3*^+/D801Y^ mice had clearly superior performance on the rotarod (latency to fall). Many were able to stay on for a full 3 min despite acceleration from 4 to 40 rpm. It is interesting that prior studies with *Atp1a3* mutant mice reported little if any deficit on the rotarod [heterozygote knock-out (DeAndrade et al., 2011; Sugimoto et al., 2014, 2018); I810N (Kirshenbaum et al., 2013, 2014); D801N on the 6N background (Hunanyan et al., 2015, 2021; Uchitel et al., 2021)]. The only *Atp1a3* mice with clear rotarod deficits were E815K (Helseth et al., 2018). How is it possible that performance is superior here, while not detected in other studies? It is unlikely that better performance corresponds to higher baseline activity in the open field (Fig. 6*A*), because all of the *Atp1a3* mouse strains, except the obviously impaired E815K mouse, exhibited higher open-field activity, while none showed better rotarod performance except very transiently in an early study of the Kawakami heterozygote knock-out (Ikeda et al., 2013). Other factors can influence rotarod performance as well. Different inbred mouse strains (technically wild type) such as C57BL/6N, FVB/N, and DBA/2 show significant differences in rotarod performance (Eltokhi et al., 2021). The possibility of a cognitive role in rotarod performance is not widely considered, but there are some examples. Conditional deletion of a transcription factor gene solely in the forebrain increased rotarod performance (Poirier et al., 2007), as did an autism-related microdeletion (Lynch et al., 2020), although in neither case to the dramatic magnitude seen here. Such studies demonstrate a role for cognitive functions in influencing behavior on the accelerating rotarod.

The *Atp1a3*^+/D801Y^ mice just keep walking forward as the rod accelerates. The rotarod is not very challenging in terms of motor coordination, since it does not require the adaptation of foot position that leads to hindlimb slipping on the elevated beam. We can speculate that the WT mice fall sooner because they are more likely to try something different to escape from the task and lose their footing. Alternatively, the *Atp1a3*^+/D801Y^ mice may have more fear of falling off, although there is no experimental support for this hypothesis. Tests of anxiety in various *Atp1a3* mouse strains, such as the elevated plus maze or center avoidance, have shown reduced, rather than elevated, levels (Kirshenbaum et al., 2011b; Ikeda et al., 2013; Sugimoto et al., 2014, 2018; Hunanyan et al., 2015; Holm et al., 2016). Lower anxiety may, however, result in steadier rotarod performance.

A more testable and specific hypothesis is that the *Atp1a3*^+/D801Y^ rotarod performance might be due to perseveration, a maladaptive cognitive manifestation. Perseveration is a deficit in the ability to change a behavior in response to changed circumstances or cues, and it sometimes manifests as an inability to stop an activity. In a broader sense, it is an impairment of behavioral flexibility, and often reported in autism. Perseveration is mentioned in several AHC case reports (Polanowska et al., 2018; Torres et al., 2018; Jasien et al., 2019; Zuniga-Ramirez et al., 2019). In mice, perseveration is a trait that coexists with hyperactivity in a fragile X mouse model (Kramvis et al., 2013), and it has been investigated in a model of Xp22.3 deletion associated with ADHD and autism (Trent et al., 2012). Perseveration may also account for the improved rotarod performance in mice modeling the autism-associated 16p11.2 Del^m^ deletion mentioned above (Lynch et al., 2020). Until the D801Y mouse's rotarod performance is explained, the possibility of improved performance should be borne in mind when interpreting preclinical testing in any *Atp1a3* mice using the rotarod, since a treatment might affect both motor deficits and cognitive features.

Another novel finding was hyperactivity during forced swimming of the D801Y mouse, which greatly reduced the time spent resting. Speculatively, this could also be a manifestation of perseveration. Hyperlocomotion has occasionally been recognized as a confounding effect in stressed animals in the Porsolt test of antidepressants (Bogdanova et al., 2013). There are also related prior observations in *Atp1a3* mice. There is the possibility that it may represent a mania-like behavior, as already described by other criteria in the I810N mice (Kirshenbaum et al., 2011a). The I810N mice also showed more time and a longer path length to reach a visible platform in the Morris water maze, without an effect on speed or floating (Kirshenbaum et al., 2015). It was thought to be mostly due to swimming at the perimeter (thigmotaxis). Similar observations on the D801N mouse led the authors to distrust the Morris water maze as a memory tool for this mutation (Hunanyan et al., 2015).

Significantly, forced swimming resulted in delayed recovery, abnormal limb positions, and severe tremor in *Atp1a3*^+/D801Y^ mice. This can be compared with the hypothermia-induced dystonia test introduced for the same mouse on the C57BL/6J background (Isaksen et al., 2017) where the same effects during recovery were elicited by hypothermia conditions alone. In the present case, strenuous swimming at room temperature may have sustained body temperature but reduced blood glucose levels, so to explore the effect of cold, we also performed forced swimming for 15 min in water at 16°C. This protocol produced the same manifestations during the recovery phase. Symptoms of *Atp1a3*^+/D801Y^ mice on the C57BL/6N background appear to be somewhat less severe than those reported on the C57BL/6J background because we did not observe convulsive behavior after the cold water swim. In one of the studies mentioned above (Isaksen et al., 2017), epileptic activity was ruled out, and abnormal limb position was demonstrated to be dystonic by electromyographic recordings of cocontraction of opposing muscles. In vivo electrophysiological recordings of activity in Purkinje neurons and deep cerebellar nuclei revealed irregular firing (a higher coefficient of variation) at baseline, and high frequency bursts during attacks (Isaksen et al., 2017), very similar to what was seen in a dystonic mouse model with mutation in *Lamb1* (Liu et al., 2015). This is evidence that dystonia, a key symptom of both RDP and AHC, can be studied in some of the *Atp1a3* mutant mice.

In summary, unstressed *Atp1a*3^tm1Ling/+^ mice have a modest reduction of Na,K-ATPase protein and activity and few symptoms. The *Atp1a3*^+/D801Y^ mice displayed new and surprising abnormalities that are quite easy to measure. Our findings support the conclusion that the *Atp1a3*^+/D801Y^ mouse model, like I810N, D801N, and E815K mice, is suitable for preclinical testing. There is great need for treatment and prevention for patients carrying an *ATP1A3* mutation. Preclinical testing has the potential to open up avenues to impact the wide range of neurologic symptoms associated with the broad spectrum of *ATP1A3* phenotypes in humans.

## Synthesis

Reviewing Editor: Christopher Colwell, UCLA School of Medicine Decisions are customarily a result of the Reviewing Editor and the peer reviewers coming together and discussing their recommendations until a consensus is reached. When revisions are invited, a fact-based synthesis statement explaining their decision and outlining what is needed to prepare a revision will be listed below. The following reviewer(s) agreed to reveal their identity: Mohamad Mikati.

The manuscript largely focuses on extending the phenotypic characterization of the already published Atp1a3<sup>tm1.1Tmklh</sup> (Atp1a3<sup>+/D801Y</sup>) and Atp1a3<sup>tm1Ling</sup> (Atp1a3<sup>+/-</sup>) mutant mouse lines. Each mutant line is analysed separately, using different tests, such that it is not possible to compare one line with the other. Some new observations within each line are reported. The manuscript would benefit from a more detailed justification for each experiment conducted, elucidating the specific reasons and hypotheses driving these investigations. It would also benefit from the authors addressing the following issues.

MAJOR POINTS

1. Atp1a3<+/tm1Ling> (MGI 3696954) and Atp1a3<+/tm1.1Kwk> (MGI 5572809) mice are both referred to as "Atp1a3<sup>+/-</sup>" or "knockout" and grouped together as though they are identical (e.g., Table 3). This is potentially misleading because the alleles are quite different. Atp1a3<tm1Ling> has a point mutation in Atp1a3 intron 4, adjacent to the exon-intron splice site, that results in aberrant splicing, adding 126 bp to the transcript. Atp1a3<tm1.1Kwk> has deletion of Atp1a3 exons 2-6. I think it is reasonable to refer to Atp1a3<+/tm1.1Kwk> mice as Atp1a3<sup>+/-</sup> or heterozygote knockout because deletion of five 5' exons is likely to result in no gene product (null allele). By contrast, Moseley et al. (2007) reports that homozygous Atp1a3<tm1Ling> show reduced Na,K-ATPase α3 isoform mRNA expression in the foetal (E18.5) brain by Northern blot. The manuscript itself reports that "Atp1a3+/- [Atp1a3<+/tm1Ling>] mice exhibited much higher α3 protein levels than the theoretical 50% level: an average of 82% (p <0.0001, n = 20) (Fig. 2A)." Referring to Atp1a3<+/tm1Ling> mice as "Atp1a3+/-" or heterozygote knockout is thus inaccurate since Atp1a3<tm1Ling> is clearly not a null allele.

2. The manuscript correctly cites Moseley et al. (2007) in stating that the aberrant transcript [with an additional 126 bp] does not produce a protein product in Atp1a3<+/tm1Ling> mice (Moseley et al., 2007). However, Figure 1A (Northern blot) in Moseley et al. (2007) shows a single Atp1a3 mRNA band in homozygous Atp1a3<tm1Ling> foetal brain, which is the same size as the band in WT controls. Figure 1B (RT-PCR) in Moseley et al. (2007) shows a WT-sized band in addition to the longer, mutant band (with an extra 126 bp) in homozygous Atp1a3<tm1Ling> foetal brain. Thus the Atp1a3<tm1Ling> allele appears to express some WT Atp1a3 transcript. Unfortunately, Moseley et al. (2007) did not test for the presence of Na,K-ATPase α3 protein in homozygous Atp1a3<tm1Ling> foetal brain by Western blotting. Nonetheless, the presence of WT Atp1a3 transcripts may explain why α3 protein levels in Atp1a3+/- [Atp1a3<+/tm1Ling>] mice were higher than the theoretical 50% level. It would be helpful if the authors could fill the gap left by Moseley et al. (2007) by performing Western blotting on homozygous Atp1a3<tm1Ling> foetal brain to test for the presence of Na,K-ATPase α3 protein.

3. Figure 2A is referenced in the text in relation to α3 protein levels in Atp1a3+/- mice and in Atp1a3+/D801Y mice, and gives P values of <0.0001 for both mouse lines. However, Figure 2A itself shows a bar chart with 'ns'. This is potentially confusing to the reader. It would be clearer if Figure 2A actually showed the significant differences referred to in the text. In the legend, could the authors better explain how an amount of protein corresponding to equal activity was loaded on the α3 subunit Western blot (Figure 2C)?

4. The manuscript describes novel methods to measure grip strength using a wire hanging traverse task, and tremor amplitude and frequency using an iPhone with LiftPulse software. However, the descriptions are currently inadequate. It would help if photographs and/or diagrams could be provided to allow others to replicate these tests. Moreover, the manuscript states that ten consecutive measurements [of post-swim tremor] per mouse were made. How did that work - were the mice exposed to 15-minute swimming sessions for 10 consecutive cycles?

5. The authors report a novel abnormality in unstressed Atp1a3+/- mice at baseline: repeated brief interruptions during voluntary running in the dark. However, the numbers of mutant and WT mice used to quantify this phenotype were very low (N=2/genotype) for mouse behavioural testing. For other motor experiments, N=6 per group were used. How reliable is the interruption data when it's based on such low numbers? Why weren't the infrared videos submitted with the manuscript? Ideally, a power analysis would be performed.

6. The manuscript mentions that Atp1a3+/D801Y mice may have more fear of falling off the rotarod, and they discusses this in relation to fear conditioning in I810S [I810N] and D801N mice. However, fear conditioning is a measure of associative learning that is motivated by fear but is not a measure of fear. Therefore, this comparison is not particularly relevant.

MINOR POINTS

7. Nitric oxide experiments are mentioned in the Materials and Methods, but not anywhere else.

8. In the description of the trisection of the brain, it would be helpful if approximate distances from bregma could be given, possibly in reference to the Allen Reference Atlas - Mouse Brain.

9. According to Mouse Genome Informatics, the correct name of Atp1a3<sup>Tm1Klh</sup> is Atp1a3<sup>tm1.1Tmklh</sup>, and the MGI ID for Atp1a3<sup>em3Lutzy</sup> is 6438218.

10. The correct name of the C57Bl/6Cr substrain of C57BL/6 is C57BL/6NCr, according to https://www.criver.com/products-services/research-models-services/nci-grantee-program.

11. In their interpretation of the ethanol stress response in Atp1a3+/- mice, the authors should consider referencing papers that describe how ethanol consumption affects in vivo Na+/K+ ATPase activity (e.g., PMID: 20520600).

12. The Myk mutant mouse line is incorrectly referred to throughout the manuscript as I810S, but it should be I810N.

13. The 'Jackson Laboratories' should be the Jackson Laboratory.

14. It is stated that '50% of the total is from the good allele', but I think it's better to refer to the wild-type or WT allele.

15. In the FVB strain, the retinal degeneration allele is called 'rd1' not 'd1', and the gene is 'Pde6b' not 'Pde6br'.

16. In Figure 1B, it is unclear what the colors of the individual data points refer to. Figure 1A suggests that light blue is WT and beige is het. If this is true for Figure 1B, it would indicate that hets weighed more than WT, contradicting what it says for Atp1a3+/D801Y males in the text. Also, could the numbers of mice of each sex/genotype (for Figure A and B) be provided in the legend?

17. The Figure 5 legend refers to B-E but the panels only go up to C.

18. The legend for Video 2 refers to reduced posture in Atp1a3+/D801Y heterozygotes. I think 'lower posture' is a better description.

19. In the rotarod test, did the performance of either genotype improve with the additional testing on days 7, 30 and 90, which would indicate motor learning?

20. In Figure 1A, the ages of the weighed mice range from 4 months to 6 months. This is a broad range. Males at 4 months might weigh less than males at 6 months, as suggested by the data in Figure 1B.

21. For the Western blot in Figure 2C, the authors should show the loading control. Also, could the authors check the size of the band. XVI-F9G10 typically gives a band at ~110 kDa.

22. The Materials and Methods should better describe the ketamine experiment. It is inadequate just to say that all mice had prior experience in the activity chamber.

23. The Materials and Methods should describe the retinal ganglion cell analysis.

24. The Discussion mentions more than a dozen LoF candidates in ClinVar, but the authors could also consider looking up ATP1A3 in gnomAD https://gnomad.broadinstitute.org/.

25. The mouse gene symbol is <i>Atp1a3</i> but it is also given in the manuscript as ATP1a3 or Atp1A3 and sometimes not in italics.

26. 'Isaksen et al (Isaksen et al., 2017)' should be 'Isaksen et al. (2017)'.

27. Figure 6 legend: '2way ANOVA' should be '2-way ANOVA'.

In this article the authors study two mouse models of functional Atp1a3 mutations: knockout, Atp1a3+/-, and a knock-in model expressing Atp1a3+/D801Y using conventional, modified, and novel behavioral testing methods. They compare these two models to each other and to other models studied in the literature. They find that these two models differ not only from each other but also from other models that express the common human mutations. There are a number of issues that need to be addressed.

1. A major point the authors need to emphasize further is that the two models do not represent the vast majority of human disease related to ATP1A3 mutations. Their first mutant models haploinsufficiency, which may presumably be present in some cases of RDP (PMID: 22842232) while their second mutant models a very rare human mutation.

2. It would be helpful, for proper phenotyping, to perform telemetry studies on their two mutant models by using implanted electrodes, video recordings and automatic seizure detection to clearly demonstrate the presence or absence of unrecognized seizures since epilepsy and seizures are such an important part of ATP1A3 related disease in humans and in other Atp1a3 models.

3. Also related to complete phenotyping: It would be helpful to perform ECG studies on these mice and for the authors to discuss that differences in mortality may potentially be related to differences in the effect of the different mutations on the heart since patients have mutation- dependent varying effects on heart rhythms (PMID: 34459253, 32913013, 26297560) and since mutant mice carrying the D801N mutation also show abnormal cardiac rhythms (PMID: 32913013). It is also important to discuss that the differences in mortality, alternatively or additionally, may also be related to the differential effects of different mutations on brains stem nuclei and on GABAergic interneurons (PMID: 29889309, 28465228) and/or on protein folding (PMID: 31425744).

4. It is very well known that the α3 subunit is inhibited by μM concentrations of ouabain while the α1 by mM concentrations. However, depending on the setup, optimizing which exact concentration to use, within each concentration range for each isoform activity, can depend on the conditions used in the specific experiment. How did they optimize the concentration of 10 μM ouabain of the α3 and the 3 mM for the α1?

5. In the discussion about ATPase activity in Figure 2 they should discuss the possibility that a dominant negative effect of the mutation/s may exist and may explain the differences in ATPase activity despite similar protein levels in the two genotypes they studied. This, also, has been demonstrated in prior studies performed on different more common mutations of this gene (PMID: 31425744) and, thus, may potentially apply to their two mutants too.

6. They should discuss the possible mechanisms and consequences of the absence of observed behavioral seizures in the two mouse models. Specifically, this could be contributing to the better performances and better survival they observed as compared to the other mice expressing the common mutations of D801N and E815K and the less frequent mutation of I810N.

7. Mechanisms of improved rotarod test and increased activity in the forced swim test are very intriguing and warrant further discussion. Some of that may represent mania like behavior and potential mechanisms to be investigated for that could include alterations in monoaminergic tone which have been related to performances on in rotarod test and on the forced swim test (PMID: 21208059, 31763490, 22025725). Similarly, resistance to ketamine may be related to NMDA receptor changes or to monoaminergic tone changes or to both (PMID: 23123346).

8. Many of the PMIDs mentioned above are for references that are already in their references. However, many others are not included in the manuscript. The article would be strengthened if the references mentioned above and that were not already cited in the submitted version, are included in a revision while addressing the points raised above.
